# 1,3,6-Trigalloylglucose: A Novel Potent Anti-*Helicobacter pylori* Adhesion Agent Derived from Aqueous Extracts of *Terminalia chebula* Retz

**DOI:** 10.3390/molecules29051161

**Published:** 2024-03-05

**Authors:** Ling Ou, Zhixiang Zhu, Yajie Hao, Qingwei Li, Hengrui Liu, Qingchang Chen, Chang Peng, Chuqiu Zhang, Yuanjing Zou, Junwei Jia, Hui Li, Yanhua Wang, Bingmei Su, Yuqian Lai, Meiyun Chen, Haobo Chen, Zhong Feng, Guimin Zhang, Meicun Yao

**Affiliations:** 1 School of Pharmaceutical Sciences (Shenzhen), Sun Yat-sen University, Shenzhen 518107, China; 2 School of Medicine and Pharmacy (Qingdao), Ocean University of China, Qingdao 266003, China; 3 International Pharmaceutical Engineering Lab of Shandong Province, Linyi 273400, China; 4 Regenerative Medicine Research Center, Future Homo Sapiens Institute of Regenerative Medicine Co., Ltd., Guangzhou 510535, China; 5 Department of Biomedical Engineering, College of Design and Engineering, National University of Singapore, Singapore 117575, Singapore; 6 Lunan Pharmaceutical Group Co., Ltd., Linyi 276006, China

**Keywords:** 1,3,6-Trigalloylglucose, *Terminalia chebula* Retz, *Helicobacter pylori*, adhesion, minimum inhibitory concentration

## Abstract

1,3,6-Trigalloylglucose is a natural compound that can be extracted from the aqueous extracts of ripe fruit of *Terminalia chebula* Retz, commonly known as “*Haritaki*”. The potential anti-*Helicobacter pylori* (HP) activity of this compound has not been extensively studied or confirmed in scientific research. This compound was isolated using a semi-preparative liquid chromatography (LC) system and identified through Ultra-high-performance liquid chromatography–MS/MS (UPLC-MS/MS) and Nuclear Magnetic Resonance (NMR). Its role was evaluated using Minimum inhibitory concentration (MIC) assay and minimum bactericidal concentration (MBC) assay, scanning electron microscope (SEM), inhibiting kinetics curves, urea fast test, Cell Counting Kit-8 (CCK-8) assay, Western blot, and Griess Reagent System. Results showed that this compound effectively inhibits the growth of HP strain ATCC 700392, damages the HP structure, and suppresses the Cytotoxin-associated gene A (Cag A) protein, a crucial factor in HP infection. Importantly, it exhibits selective antimicrobial activity without impacting normal epithelial cells GES-1. In vitro studies have revealed that 1,3,6-Trigalloylglucose acts as an anti-adhesive agent, disrupting the adhesion of HP to host cells, a critical step in HP infection. These findings underscore the potential of 1,3,6-Trigalloylglucose as a targeted therapeutic agent against HP infections.

## 1. Introduction

*Helicobacter pylori* (HP), a gram-negative, microaerophilic, spiral-shaped, and flagellated bacterium, has gained widespread notoriety due to its infection of nearly half of the global population, with the human stomach being its primary reservoir [1,2]. The prevalence of this infection varies significantly based on geographic location, age, ethnicity, and socioeconomic status, with a notably higher occurrence in developing countries and underprivileged communities [3]. HP demonstrates remarkable microbiological resilience, enabling it to withstand even the harshest of conditions, including the acidic environment of the stomach. The transmission of this infection primarily occurs through the oral–fecal route [4], particularly via contaminated water [5] and food [6], and there is potential for oral–oral transmission as evidenced by the bacterium’s isolation in saliva and dental plaque [3,7,8,9,10].

The impact of HP infection is significant, as it is the primary cause of chronic gastritis and peptic ulcer disease [11]. Additionally, this bacterium plays a crucial role in the pathogenesis of distal gastric adenocarcinoma and gastric mucosa-associated lymphoid tissue (MALT) lymphoma [12,13]. Its insidious influence on gastric cell proliferation, combined with an inadequate balance of apoptosis, contributes to gastric carcinogenesis. The spectrum of gastroduodenal diseases associated with the progression of HP infection is vast and varied, leading to diverse clinical outcomes. The interaction of bacterial virulence factors, environmental influences, and the genetic composition of the host likely contributes to the diverse gastric phenotypes and clinical consequences associated with HP infection. Intriguingly, reports have also emerged regarding the association between HP infection and extra-gastric diseases [14,15], adding complexity and debate to this already intricate topic. The literature outlines numerous extra-digestive diseases potentially linked to HP infection, spanning neurological, dermatological, hematologic, ocular, cardiovascular, metabolic, and allergic conditions [15,16,17,18]. While some associations lack a clear explanation of the pathogenic mechanism, others exhibit a strong correlation, prompting guidelines for the treatment of HP infection to recommend determination and eradication therapy in these circumstances [19]. Overall, the implications of HP infection extend far beyond the digestive system, presenting a complex array of potential health consequences that continue to engage scientific inquiry and clinical attention.

The escalating antibiotic resistance of HP has compromised the effectiveness of commonly used antimicrobial agents such as clarithromycin, levofloxacin, and metronidazole, often leading to failure in eradicating the infection [20,21,22,23,24]. Research has indicated that the global resistance of HP to antibiotics has reached alarming levels, significantly undermining the efficacy of treatment [25]. This resistance poses a significant challenge to the standard antimicrobial agents utilized in HP treatment, and it raises concerns about the long-term efficacy of current therapeutic approaches. Consequently, there is an urgent need for the development of novel strategies and alternative treatment modalities to effectively address the challenge posed by antibiotic-resistant HP strains and to improve the overall management of HP infections.

Increasing evidence supports the efficacy of traditional Chinese medicines (TCMs) in treating various diseases. Reviews evaluating the effectiveness and safety of TCMs for H. pylori treatment propose their potential as an alternative therapy for HP infection [26,27,28]. TCM has demonstrated a reduced likelihood of inducing bacterial resistance, offering a potential advantage over conventional antibiotics in addressing HP infection. Moreover, TCM is derived from natural sources, thereby circumventing the toxic side effects associated with chemical drugs and presenting a safer treatment alternative. Additionally, TCM interventions for HP-related gastric diseases often manifest dual therapeutic and regulatory effects, with unique formulas specifically targeting affected areas, expelling pathogenic factors, and yielding enduring efficacy. These findings suggest that TCM may present promising alternatives to conventional antibiotic therapies, particularly in scenarios where antibiotic resistance poses a significant challenge to effective treatment.

In our recent investigation, we discovered that certain traditional Chinese medicines (TCMs) demonstrate activity against HP, including *Terminalia chebula* Retz [29]. However, the active compound responsible for this activity has not yet been fully characterized. Therefore, our current research aims to identify the novel anti-HP component present in *Terminalia chebula* Retz. Our initial exploration has unveiled 1,3,6-Trigalloylglucose, obtained from the aqueous extract of *Terminalia chebula* Retz, as a promising in vitro anti-HP agent. Furthermore, our initial findings suggest that 1,3,6-Trigalloylglucose acts as an in vitro anti-HP adhesion agent, effectively inhibiting HP activity.

## 2. Results

### 2.1. Identification of Compound ***3***

**Figure 1:**
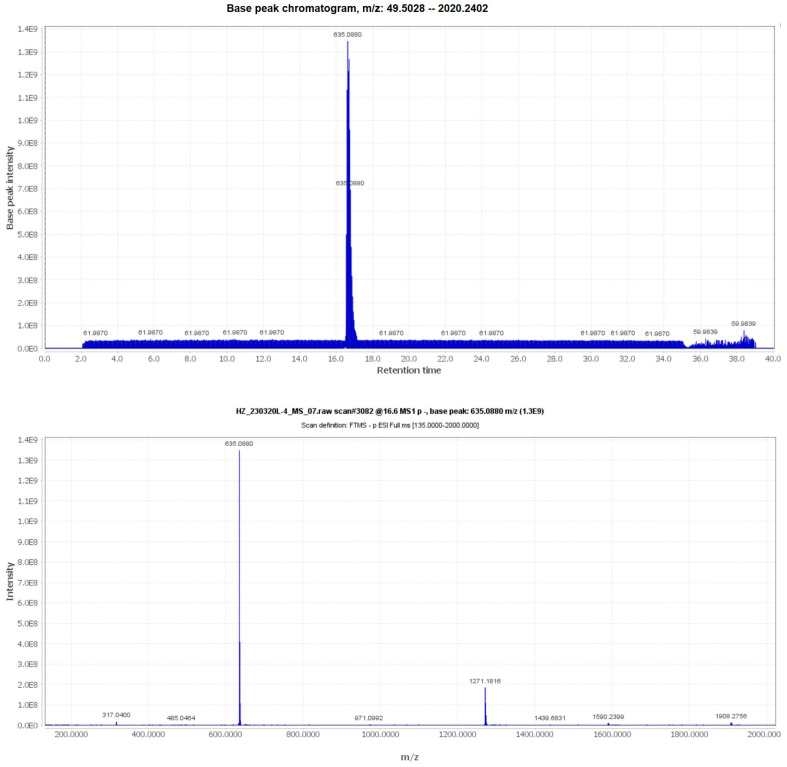
The UPLC-MS/MS information of compound **3**.

**Figure 2:**
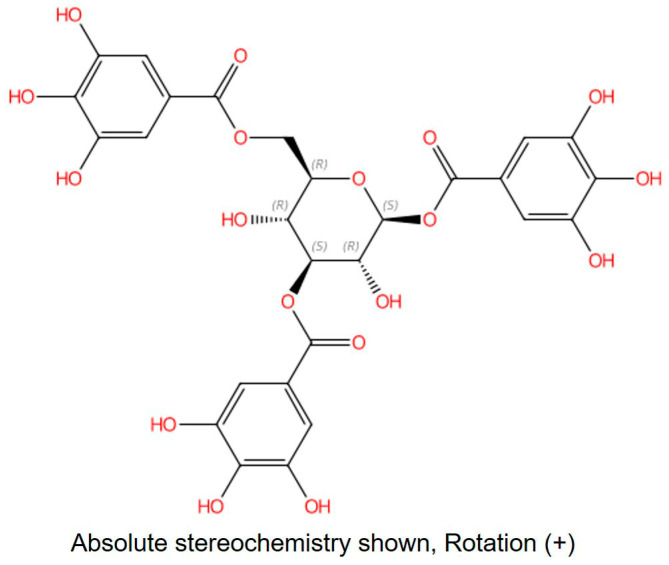
The structure of compound **3**. CAS: 18483-17-5; CAS Name: 1,3,6-Tri-*O*-galloyl-β-d-glucose.

The m/z information of compound **3** was identified as 636.0880 in negative ion mode, as illustrated in Figure 1. To delve deeper into the structural characterization, NMR analysis was performed. Based on the information presented in Figure S1 and Table 1, along with references [30,31,32], compound **3** was unequivocally identified as 1,3,6-Trigalloylglucose. The CAS Registry Number is 18483-17-5, and its CAS Name is 1,3,6-Tri-O-galloyl-β-D-glucose (Figure 2).

### 2.2. 1,3,6-Trigalloylglucose Possessed Anti-HP Activity Rather than Bactericidal Activity

To investigate the impact of 1,3,6-Trigalloylglucose on HP, MIC and MBC tests were performed. As seen in Table 2, the MICs of 1,3,6-Trigalloylglucose range from 16 to 128 µg/mL, while the MBC is greater than eight times the MIC, indicating that 1,3,6-Trigalloylglucose exerts bacteriostatic rather than bactericidal activity against HP.

### 2.3. 1,3,6-Trigalloylglucose Significantly Damaged the Bacterial Structure

**Figure 3:**
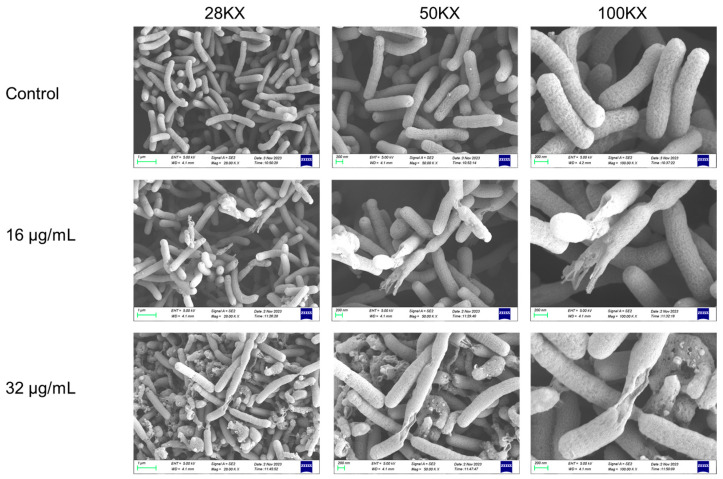
The scanning electron microscopy (SEM) images of the structure of HP strain ATCC 700392 after 1,3,6-Trigalloylglucose treatment for 24 h.

To further investigate 1,3,6-Trigalloylglucose’s impact on bacterial structure, we conducted SEM experiments. As shown in Figure 3, the surface of the bacteria was slightly damaged after being treated with 16 μg/mL 1,3,6-Trigalloylglucose for 24 h. With an increase in drug concentration to 32 μg/mL, both the quantity and area of damaged bacteria enlarged. These results revealed that 1,3,6-Trigalloylglucose significantly disrupted the bacterial structure, leading to bacterial rupture.

### 2.4. 1,3,6-Trigalloylglucose Inhibited the Growth of HP Strain ATCC 700392

**Figure 4:**
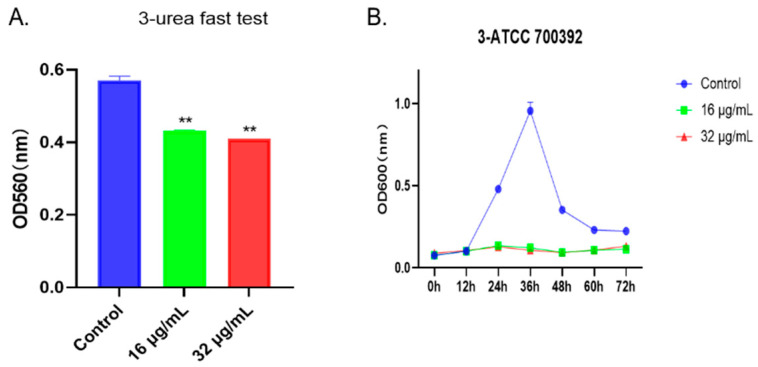
1,3,6-Trigalloylglucose inhibited the growth of HP strain ATCC 700392. (**A**) Urea fast test. (**B**) The growth curve of ATCC 700392. ** represents compared with control group, *p* < 0.01.

To visually assess the impact of the drug on bacteria, we employed the rapid urease test and the agar diffusion method. As shown in Figure 4A, after treatment with 1,3,6-Trigalloylglucose at concentrations of 16 µg/mL or 32 µg/mL, the urease activity was significantly inhibited. Additionally, the growth curve results indicated a significant inhibition of bacterial growth at concentrations of 16 µg/mL or 32 µg/mL from 24 h to 72 h (Figure 4B). Taken together, the growth of the HP strain ATCC 700392 was inhibited by 1,3,6-Trigalloylglucose.

### 2.5. 1,3,6-Trigalloylglucose Repressed the Cag A Protein

**Figure 5:**
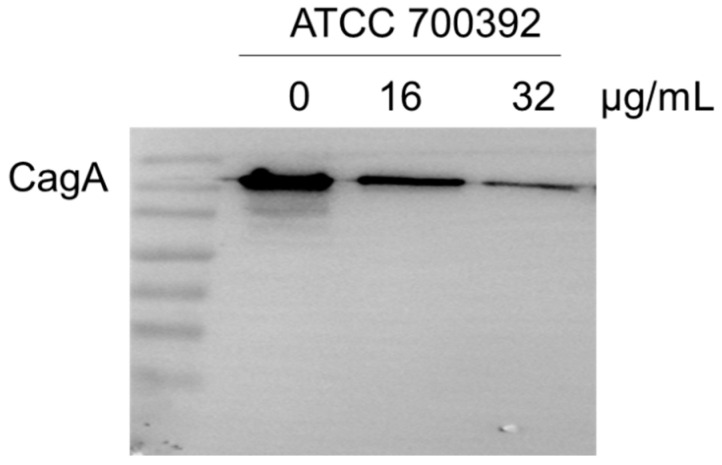
1,3,6-Trigalloylglucose repressed the Cag A protein. ATCC700392 was treated with different doses of 1,3,6-Trigalloylglucose (0, 16, and 32 µg/mL) for 24 h.

Cag A protein is a pivotal factor in HP infection. To investigate the effect of 1,3,6-Trigalloylglucose on Cag A expression, we have determined it by Western blot. The results showed that 32 µg/mL 1,3,6-Trigalloylglucose significantly inhibits the expression of the virulence factor Cag A (Figure 5).

### 2.6. 1,3,6-Trigalloylglucose Has No Significant Impact on Normal Epithelial Cells GES-1

**Figure 6:**
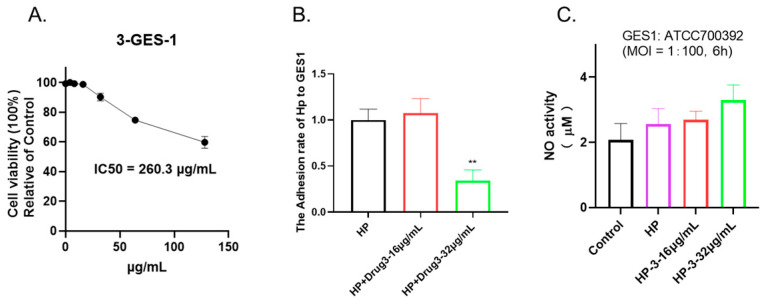
1,3,6-Trigalloylglucose acts as an anti-adhesive agent in vitro. (**A**) Cell viability. GES-1 cells were treated with different doses of 1,3,6-Trigalloylglucose (0–128 µg/mL) for 24 h. (**B**) Anti-adhesive activity. (**C**) NO activity. The GES-1 cells were co-cultured with HP at a ratio of 1:100, along with different doses of 1,3,6-Trigalloylglucose (0, 16, 32 µg/mL) for a duration of 6 h. ** represents compared with HP group, *p* < 0.01.

To evaluate the cytotoxicity of 1,3,6-Trigalloylglucose, cell viability was determined by CCK-8 assay. As shown in Figure 6A, under 64 µg/mL 1,3,6-Trigalloylglucose has no significant impact on normal epithelial cells GES-1, which indicates its safety in this regard, despite its antibacterial properties.

### 2.7. 1,3,6-Trigalloylglucose Acts as an Anti-Adhesive Agent In Vitro but Does Not Affect No Activity

To further investigate its potential effects, we co-cultured cells and bacteria and treated them with 1,3,6-Trigalloylglucose for 6 h. Our findings indicate that 1,3,6-Trigalloylglucose at a concentration of 32 µg/mL significantly inhibited bacterial adhesion to the cells. This suggests that it can serve as an antibacterial adhesion agent (Figure 6B). In addition, we also explored whether it has anti-NO effects and found that it does not affect NO activity (Figure 6C).

## 3. Discussion

1,3,6-Trigalloylglucose was suggested to have a wide range of therapeutic applications, encompassing anti-inflammatory, anti-diabetic, and antiviral properties. This suggests its potential as a versatile therapeutic agent with diverse clinical applications. Derived from the AcOEt extract of the seeds of *Picrorhiza kurroa*, 1,3,6-Trigalloylglucose, a phenolic compound, has demonstrated inhibition of cyclooxygenase-1 (COX-1) [30]. Furthermore, it has been identified in Black Walnut Kernels [33] showcasing potential health-promoting activities. Additionally, 1,3,6-Trigalloylglucose from Turkish galls has been acknowledged as a potential α-glucosidase inhibitor [34]. A recent study has also revealed its potential as an inhibitor for variants of SARS-CoV-2 [35].

Our research has marked a significant milestone as the first to demonstrate the anti-*Helicobacter pylori* (HP) activity of 1,3,6-Trigalloylglucose derived from aqueous extracts of *Terminalia chebula* Retz. Based on electron microscopy results, it has been observed to cause substantial damage to the structure of HP bacteria, resulting in structural damage and fracturing. Moreover, at a concentration of 32 µg/mL, it has notably inhibited bacterial adhesion to the GES1 cells. Consequently, we hypothesize that while it may not function as a bactericidal agent, it holds potential as an antibacterial agent, particularly in impeding bacterial adhesion.

Most notably, 1,3,6-Trigalloylglucose has proven its safety profile by exhibiting no toxic side effects on normal cells at the concentration required for its antibacterial effect. Building upon our previous studies that established the robust antimicrobial properties of *Terminalia chebula* Retz, our current research has identified a novel active ingredient within its aqueous extracts. This compound acts as an antimicrobial agent by functioning as an anti-adhesive agent. While its bactericidal effect may not match that of the entire aqueous extracts of *Terminalia chebula* Retz, we speculate that this plant may harbor additional components contributing to its overall antimicrobial activity. The discovery of this new active ingredient in the aqueous extracts of *Terminalia chebula* Retz presents an exciting opportunity for further exploration. It not only underscores the complexity of the plant’s therapeutic potential but also invites future research endeavors to identify and characterize additional components that contribute to its antimicrobial activity. This could potentially unveil novel compounds with diverse therapeutic applications.

This groundbreaking discovery holds tremendous promise for the development of innovative antibacterial treatments. Not only does it underscore its potential in combating *Helicobacter pylori* (HP) infections, but it also paves the way for exploring its effectiveness against a spectrum of other bacterial strains. Moreover, its favorable safety profile enhances its allure as a potential therapeutic agent. Subsequent investigations will focus on elucidating the underlying mechanisms of action of 1,3,6-Trigalloylglucose, as well as exploring potential synergistic effects with other compounds present in *Terminalia chebula* Retz. Additionally, imperative clinical studies to assess its therapeutic efficacy and safety in human subjects will be essential for transitioning these findings into practical medical applications.

## 4. Materials and Methods

### 4.1. Reagents

Columbia agar base and brain–heart infusion (BHI) were obtained from Oxoid Ltd. (Basingstoke, Hants, UK). The sterile-defibrinated sheep blood was acquired from Hongquan Biotechnology (Guangzhou, China). Gibco-life Technologies LLC. (Rockville, MD, USA) supplied phosphate-buffered saline (PBS), Roswell Park Memorial Institute (RPMI) 1640 medium, and fetal bovine serum (FBS). Beyotime (Shanghai, China) provided RIPA reagent, PMSF, phosphatase inhibitor cocktail (50×), sodium dodecyl sulfate–polyacrylamide gel electrophoresis (SDS-PAGE) Gel Quick Preparation Kit, BeyoColor^TM^ Prestained Color Protein Marker, BeyoECL Star Ultrasensitive Chemiluminescence Kit, and the second antibodies against rabbit-HRP and mouse-HRP. Additionally, the protease inhibitor cocktail was sourced from Roche (Basel, Switzerland) and Anti-HP CagA, and the m-IgG Fc BP-HRP from Santa Cruz (Dallas, TX, USA).

### 4.2. Terminalia chebula Retz Aqueous Extract Preparation

The mature fruit of *Terminalia chebula* Retz (Yunnan, China, Guangzhou Zhining Pharmaceutical Co., Ltd., Guangzhou, China, Lot No. 210901) was purchased, authenticated, and classified as *Terminalia chebula* Retz’s mature fruit by the Chief Pharmacist, Weixing Zhu [29]. A total of 20 g of mature fruit of *Terminalia chebula* Retz underwent extraction with 10-fold double-distilled water, was boiled three times at 90 °C for 1 h, spin-concentrated, freeze-dried, and finally stored at −20 °C, which was previously described [29].

### 4.3. The Preparation Process of the Compound by Semi-Preparative LC System

The 5 g water extract of *Terminalia chebula* Retz was dissolved in 170 mL of water, filtered using a 0.22 µm filter, and utilized as the sample. The semi-preparative LC system was employed to separate and prepare the sample under specific chromatographic conditions. The column used was YMC Actus Triart C18, 20 × 250 mm, with 5 µm particle size. The mobile phase consisted of A: 0.1% formic acid water and B: acetonitrile. The gradient conditions were as follows: 0 min: 95% A, 5% B; 50 min: 86% A, 14% B; 80 min: 76% A, 24% B; 80.10 min: 0% A, 100% B; 85 min: 0% A, 100% B; 85.1 min: 95% A, 5% B; and 90 min: 95% A, 5% B. The detector operated at 270nm with a flow rate of 16 mL/min. The compound collected in the No. 19 tube during the 58-min peak was identified as Compound **3** and subsequently freeze-dried for structural characterization.

### 4.4. Ultra-High-Performance Liquid Chromatography-MS/MS (UPLC-MS/MS)

The test sample fraction, Compound **3**, was analyzed using liquid chromatography–mass spectrometry under the specified conditions. The column employed was YMC Triart C18, 2.1 × 100 mm, with 1.9 µm particle size. The mobile phase comprised A: 0.1% formic acid water and B: acetonitrile, with the following gradient conditions: 0 min: 95% A, 5% B; 20 min: 70% A, 30% B; 27 min: 70% A, 30% B; 27.10 min: 95% A, 5% B; and 40 min: 95% A, 5% B. The detector operated at 270nm with a flow rate of 0.2 mL/min. The monitoring mode was Full MS ddMS2, and the spray voltage was (+) 3500 V (−) 3000 V. Additionally, the auxiliary gas flow rate was 7 Arb, the auxiliary gas heater temperature was 300 °C, the sheath gas flow rate was 35 Arb, and the capillary temperature was 325 °C.

### 4.5. Nuclear Magnetic Resonance (NMR) Identification

Compound **3** was dissolved in Methanol-D4 for structural identification. The structural analysis was performed using the AVANCE NEO 600M system (Bruker BioSpin GmBH; Rheinstetten, Germany).

### 4.6. HP Culture, Cell Culture, and Co-Culture

The standard HP strains ATCC 43504 and ATCC 700392, as well as GES-1 cells, were purchased from the American Type Culture Collection (ATCC, Manassas, VA, USA). CS01 was generously granted by Professor Jing Liu (University of Shanghai for Science and Technology, Shanghai, China), and QYZ-003 and QYZ-004 were obtained from the Qingyuan Hospital of Traditional Chinese Medicine (Qingyuan, China). All HP strains were authenticated, cultured, and stored at the School of Pharmaceutical Sciences (Shenzhen, China), Sun Yat-sen University. The strains were cultured on a Columbia agar base supplemented with 5% sterile defibrinated sheep blood or in BHI broth containing 10% FBS, shaken at 150 rpm, and subsequently incubated at 37 °C in a tri-gas incubator (ESCO, Singapore) with a gas composition of 10% CO_2_, 5% O_2_, and 85% N_2_ for a period of two to three days, as previously described [29]. GES-1 cells were cultured with RPMI 1640 medium containing 10% FBS. For co-culture, following overnight incubation of the GES1 cells on 24-well plates, HP was added to the cells at an MOI ratio of 100:1, along with varying concentrations of drugs, for a co-incubation period of 6 h before being used for subsequent experiments.

### 4.7. MIC Assay and Minimum Bactericidal Concentration (MBC) Assay

The MIC assays against HP were conducted using the broth microdilution method in 96-well plates, as described by Shen et al. in 2021 [36]. The experiment involved preparing two-fold dilutions of the drugs in a 96-well microtiter plate, with 50 μL per well. A 2-day-old HP strain culture grown on 5% sheep blood Columbia agar was harvested and then added to the microtiter plate, with 50 μL per well, resulting in a final concentration of 1 × 10^6^ CFU/mL. The plates were then placed in a microaerophilic atmosphere at 37 °C, with continuous shaking at 150 rpm for 3 days. After incubation, the plates were visually examined, and the MIC was determined as the lowest concentration that resulted in no turbidity, indicating inhibition of bacterial growth. To ensure the reliability of the results, clarithromycin, an antibiotic, was used as a positive control. Additionally, negative control samples dissolved in blank medium without microorganisms, and growth control samples without any test compounds were included in the experiment. Following the measurement of the MIC values, 100 μL of the drug-treated solution at concentrations 1, 2, 4, and 8 times the MIC were then cultured on Columbia agar base supplemented with 5% sheep blood.

### 4.8. Scanning Electron Microscope (SEM)

The morphology of HP was examined using SEM. Initially, the HP strain ATCC 700392 was treated with drugs, both with and without treatment, at the MIC for 24 h. Afterward, the bacteria were harvested via centrifugation at 6000 rpm for 3 min and subsequently washed twice with PBS. The samples were then treated with 2.5% glutaraldehyde and left to fix overnight at 4 °C. For initial dehydration, a graduated ethanol series was utilized before the samples underwent lyophilization and fixation. Following this, the specimens were further processed through metal spraying and subsequently observed using a scanning electron microscope (Sigma 500, ZEISS, Oberkochen, Germany), as described by a prior study [29].

### 4.9. Inhibiting Kinetics Curves

Inhibitory kinetics curves were created by exposing HP ATCC 700392 to different concentrations of drugs. The *Helicobacter pylori* strain ATCC 700392 was subjected to varying drug concentrations in brain–heart infusion (BHI) broth supplemented with 10% fetal bovine serum (FBS). The samples were subsequently agitated at 150 rpm within a tri-gas incubator. This experimental setup allowed for the assessment of the bacterial response to different drug doses in a simulated in vitro environment, providing valuable insights into the efficacy of the drugs against the HP strain. The use of BHI broth supplemented with FBS ensured a nutrient-rich growth medium, while the agitation in the tri-gas incubator mimicked physiological conditions, potentially yielding clinically relevant results. Samples of 100 μL were taken at specific time points (0, 12, 24, 36, 48, 60, and 72 h) for absorbance measurement at OD 600 nm, as previously described [29].

### 4.10. Nitric Oxide (NO) Activity

After co-culture for 6 h, cell culture supernatant was collected and NO activity was measured using the Griess Reagent System kit according to the instructions. To generate a standard curve for nitrite, standard nitrite solutions are prepared and added to a 96-well plate along with the samples. Following this, Griess reagents (sulfanilamide and N-(1-naphthyl)ethylenediamine dihydrochloride) are added. After a specified incubation period, to quantify the nitrite levels in the samples based on the standard curve, the optical density at 540 nm was measured using a microplate reader (Multiskan GO, Thermo Scientific, Waltham, MA, USA).

### 4.11. Urea Fast Test

The HP strain ATCC 700392 was exposed to drugs for 24 h, followed by the addition of urea test solutions. Then, optical density at 560 nm was measured using a microplate reader (Multiskan GO, Thermo Scientific, USA).

### 4.12. Cell Viability

Cell viability was assessed using the Cell Counting Kit-8 (CCK-8) assay (Beyotime, Shanghai, China) following the manufacturer’s instructions. A 96-well plate was seeded with 10,000 GES-1 cells and incubated overnight. Subsequently, the drug was introduced and treated for 24 h. Following this incubation period, CCK-8 solutions were applied to the 96-well plate and allowed to incubate for an additional 2 h. The optical density at 450 nm was measured using a microplate reader (Multiskan GO, Thermo Scientific, USA).

### 4.13. Cell Adhesion Activity

The GES-1 cells were co-cultured with HP at a ratio of 1:100, along with different doses of 1,3,6-Trigalloylglucose (0, 16, 32 µg/mL) for a duration of 6 h. After co-culture for 6 h, the GES-1 cells were washed twice with PBS. Then, urea test solutions were added into cells, which allowed for the assessment of urea levels of HP within the cells, and next OD560 was measured by a microplate reader (Multiskan GO, Thermo Scientific, USA).

### 4.14. Western Blot

The HP strain ATCC 700392 was treated with drugs for 24 h. After centrifugation at 6000 rpm for 3 min, the supernatant was carefully decanted, and the bacterial pellet was collected. The bacterial pellet was then resuspended in RIPA lysis buffer containing PMSF and incubated for 30 min for lysis. Following this, the lysate was centrifuged at 12,000 rpm for 10 min, and the resulting supernatant was collected and used as the protein extract for the HP strain ATCC 700392. This process allowed for the isolation of proteins for downstream analysis and experimentation. Following protein concentration detection using the BCA method, the lysed solutions were boiled with the loading buffer at 100 °C for 10 min. Subsequently, 30 μg of proteins from each group were loaded onto SDS-PAGE gels, transferred onto PVDF membranes, blocked with 5% milk, and then incubated with anti-Cag A as the primary antibody overnight at 4 °C. After washing with PBST three times for 10 min each, the membranes were incubated with the secondary antibody for 1 h at room temperature. Finally, the membranes were exposed using BeyoECL (Beyotime, China) under a Visualizer (ChemiScope 6200, Clinx Science Instruments, Shanghai, China).

### 4.15. Statistical Analysis

The data was visualized using GraphPad Prism 8.0.2 software, and statistical analysis was performed using the two-sided Student’s *t*-test or one-way ANOVA followed by an appropriate post hoc test. Statistical significance was denoted by * *p* < 0.05 or ** *p* < 0.01.

## 5. Conclusions

1,3,6-Trigalloylglucose has exhibited robust anti-*Helicobacter pylori* (HP) activity, effectively inhibiting the growth of HP strain ATCC 700392 and repressing the Cag A protein, a key factor in HP infection. Importantly, it did not impact normal epithelial cells GES-1, highlighting its selective antimicrobial activity. In vitro studies revealed that 1,3,6-Trigalloylglucose acts as an anti-adhesive agent, disrupting the adhesion of HP to host cells, a crucial step in HP infection. Additionally, it has no effect on NO activity. These findings highlight the potential of 1,3,6-Trigalloylglucose as a targeted therapeutic agent against HP infections. Future research should focus on understanding its anti-adhesive and anti-virulence properties, as well as evaluating its efficacy in relevant in vivo models. Clinical studies are crucial for assessing its safety and efficacy in humans, advancing its potential application in combating HP-related pathologies.

## Figures and Tables

**Table 1:** The information of compound **3**.

Method	Results
UPLC-MS/MS	m/z: 635.0880 [M-H]^−^
^1^H	Chemical shift(δ): 3.77 (dd, 1H, Glc H-C(2)); 3.79 (dd, 1H, Glc H-C(4)); 3.88 (ddd, 1H, Glc H-C(5)); 4.46 (dd, 1H, Glc H_a_-C(6)); 4.59 (dd, 1H, Glc H_b_-C(6)); 5.28 (t, 1H, Glc H-C(3)); 5.82 (d, 1H, Glc H-C(1)); 7.09 (s, 2 H, Gal^3^ H-C(2,6)); 7.14 (s, 2 H, Gal^2^ H-C(2,6)); 7.16 (s, 2 H, Gal^1^ H-C(2,6)).
^13^C	Chemical shift(δ): 64.27 (Glc C(6)); 69.75 (Glc C(5)); 72.67 (Glc C(4)); 76.45 (Glc C(3)); 78.97 (Glc C(2)); 95.92 (Glc C(1)); 110.65, 110.45, 110.25 (3 C(2,6) of Gal); 121.66, 121.31, 120.53 (3C(1)of Gal); 140.51, 139.94, 139.83 (3C(4)of Gal); 146.56, 146.52, 146.46 (3C(3,5) of Gal); 168.27, 168.17, 166.88 (3 C=O).

**Table 2:** The MIC and MBC of 1,3,6-Trigalloylglucose.

HP Strains	MIC (µg/mL)	MBC (µg/mL)
ATCC 700392	32	>256
ATCC 43504	32	-
CS01	128	-
QYZ003	16	-
QYZ004	32	>256

HP represents *Helicobacter pylori*; MIC represents Minimum inhibitory concentration; MBC represents minimum bactericidal concentration; *-* represents not detected.

## Data Availability

The data presented in this study are available on request from the corresponding author.

## Supplementary Materials

The following supporting information can be downloaded at: https://www.mdpi.com/article/10.3390/molecules29051161/s1, Figure S1: the information of compound **3** by NMR analysis.

## References

[1] Bravo D., Hoare A., Soto C., Valenzuela M.A., Quest A.F. (2018). *Helicobacter pylori* in human health and disease: Mechanisms for local gastric and systemic effects. World J. Gastroenterol..

[2] Salih B.A. (2009). *Helicobacter pylori* infection in developing countries: The burden for how long?. Saudi J. Gastroenterol..

[3] Kayali S., Manfredi M., Gaiani F., Bianchi L., Bizzarri B., Leandro G., Di Mario F., De’Angelis G.L. (2018). Helicobacter pylori, transmission routes and recurrence of infection: State of the art. Acta Biomed..

[4] Lindkvist P., Wadstrom T., Giesecke J. (1995). *Helicobacter pylori* infection and foreign travel. J. Infect. Dis..

[5] Aziz R.K., Khalifa M.M., Sharaf R.R. (2015). Contaminated water as a source of *Helicobacter pylori* infection: A review. J. Adv. Res..

[6] Soares G.A.S., Moraes F.A.S., Ramos A., Santiago S.B., Germano J.N., Fernandes G.A., Curado M.P., Barbosa M.S. (2023). Dietary habits and *Helicobacter pylori* infection: Is there an association?. Ther. Adv. Gastroenterol..

[7] Baker K.H., Hegarty J.P. (2001). Presence of *Helicobacter pylori* in drinking water is associated with clinical infection. Scand. J. Infect. Dis..

[8] Kheyre H., Morais S., Ferro A., Costa A.R., Norton P., Lunet N., Peleteiro B. (2018). The occupational risk of *Helicobacter pylori* infection: A systematic review. Int. Arch. Occup. Environ Health.

[9] Kotilea K., Bontems P., Touati E. (2019). Epidemiology, Diagnosis and Risk Factors of *Helicobacter pylori* Infection. Adv. Exp. Med. Biol..

[10] Kuo Y.C., Yu L.Y., Wang H.Y., Chen M.J., Wu M.S., Liu C.J., Lin Y.C., Shih S.C., Hu K.C. (2022). Effects of *Helicobacter pylori* infection in gastrointestinal tract malignant diseases: From the oral cavity to rectum. World J. Gastrointest. Oncol..

[11] Joob B., Wiwanitkit V. (2014). *Helicobacter pylori* infection, chronic kidney disease, and peptic ulcer disease. J. Chin. Med. Assoc..

[12] Park J.B., Koo J.S. (2014). *Helicobacter pylori* infection in gastric mucosa-associated lymphoid tissue lymphoma. World J. Gastroenterol..

[13] Hu Q., Zhang Y., Zhang X., Fu K. (2016). Gastric mucosa-associated lymphoid tissue lymphoma and *Helicobacter pylori* infection: A review of current diagnosis and management. Biomark. Res..

[14] Mekonnen H.D., Fisseha H., Getinet T., Tekle F., Galle P.R. (2018). *Helicobacter pylori* Infection as a Risk Factor for Hepatocellular Carcinoma: A Case-Control Study in Ethiopia. Int. J. Hepatol..

[15] Gravina A.G., Priadko K., Ciamarra P., Granata L., Facchiano A., Miranda A., Dallio M., Federico A., Romano M. (2020). Extra-Gastric Manifestations of *Helicobacter pylori* Infection. J. Clin. Med..

[16] Park A.M., Tsunoda I. (2022). *Helicobacter pylori* infection in the stomach induces neuroinflammation: The potential roles of bacterial outer membrane vesicles in an animal model of Alzheimer’s disease. Inflamm. Regen..

[17] Aramouni K., Assaf R.K., Azar M., Jabbour K., Shaito A., Sahebkar A., Eid A.A., Rizzo M., Eid A.H. (2023). Infection with *Helicobacter pylori* may predispose to atherosclerosis: Role of inflammation and thickening of intima-media of carotid arteries. Front. Pharmacol..

[18] Noori M., Mahboobi R., Nabavi-Rad A., Jamshidizadeh S., Fakharian F., Yadegar A., Zali M.R. (2023). *Helicobacter pylori* infection contributes to the expression of Alzheimer’s disease-associated risk factors and neuroinflammation. Heliyon.

[19] Kotilea K., Iliadis E., Nguyen J., Salame A., Mahler T., Miendje Deyi V.Y., Bontems P. (2023). Antibiotic resistance, heteroresistance, and eradication success of *Helicobacter pylori* infection in children. Helicobacter.

[20] Smith S.I., Yamaoka Y. (2023). Antibiotic Resistance and Therapy for *Helicobacter pylori* Infection. Antibiotics.

[21] Setshedi M., Smith S.I. (2023). *Helicobacter pylori* Infection: Antibiotic Resistance and Solutions for Effective Management in Africa. Antibiotics.

[22] Schubert J.P., Ingram P.R., Warner M.S., Rayner C.K., Roberts-Thomson I.C., Costello S.P., Bryant R.V. (2023). Refractory *Helicobacter pylori* infection in Australia: Updated multicentre antimicrobial resistance. Intern. Med. J..

[23] Malfertheiner P., Camargo M.C., El-Omar E., Liou J.M., Peek R., Schulz C., Smith S.I., Suerbaum S. (2023). *Helicobacter pylori* infection. Nat. Rev. Dis. Primers.

[24] Dascalu R.I., Bolocan A., Paduaru D.N., Constantinescu A., Mitache M.M., Stoica A.D., Andronic O. (2023). Multidrug resistance in *Helicobacter pylori* infection. Front. Microbiol..

[25] Fauzia K.A., Tuan V.P. (2024). Rising resistance: Antibiotic choices for *Helicobacter pylori* infection. Lancet Gastroenterol. Hepatol..

[26] Lin J., Huang W.W. (2009). A systematic review of treating *Helicobacter pylori* infection with Traditional Chinese Medicine. World J. Gastroenterol..

[27] Li R.J., Dai Y.Y., Qin C., Huang G.R., Qin Y.C., Huang Y.Y., Huang Z.S., Luo X.K., Huang Y.Q. (2021). Application of traditional Chinese medicine in treatment of *Helicobacter pylori* infection. World J. Clin. Cases.

[28] Zhong M.F., Li J., Liu X.L., Gong P., Zhang X.T. (2022). TCM-Based Therapy as a Rescue Therapy for Re-Eradication of *Helicobacter pylori* Infection: A Systematic Review and Meta-Analysis. Evid. Based Complement. Altern. Med..

[29] Ou L., Liu H.R., Shi X.Y., Peng C., Zou Y.J., Jia J.W., Li H., Zhu Z.X., Wang Y.H., Su B.M. (2024). *Terminalia chebula* Retz. aqueous extract inhibits the *Helicobacter pylori*-induced inflammatory response by regulating the inflammasome signaling and ER-stress pathway. J. Ethnopharmacol..

[30] Zhang Y., DeWitt D.L., Murugesan S., Nair M.G. (2004). Novel lipid-peroxidation- and cyclooxygenase-inhibitory tannins from *Picrorhiza kurroa* seeds. Chem. Biodivers.

[31] Lee D.Y., Kim H.W., Yang H., Sung S.H. (2017). Hydrolyzable tannins from the fruits of *Terminalia chebula* Retz and their alpha-glucosidase inhibitory activities. Phytochemistry.

[32] Nigam M., Mishra A.P., Adhikari-Devkota A., Dirar A.I., Hassan M.M., Adhikari A., Belwal T., Devkota H.P. (2020). Fruits of *Terminalia chebula* Retz.: A review on traditional uses, bioactive chemical constituents and pharmacological activities. Phytother. Res..

[33] Vu D.C., Vo P.H., Coggeshall M.V., Lin C.H. (2018). Identification and Characterization of Phenolic Compounds in Black Walnut Kernels. J. Agric. Food Chem..

[34] Wang Z., Zhang Y., Yan H. (2022). In situ net fishing of alpha-glucosidase inhibitors from evening primrose (*Oenothera biennis*) defatted seeds by combination of LC-MS/MS, molecular networking, affinity-based ultrafiltration, and molecular docking. Food Funct..

[35] Binette V., Cote S., Haddad M., Nguyen P.T., Belanger S., Bourgault S., Ramassamy C., Gaudreault R., Mousseau N. (2021). Corilagin and 1,3,6-Tri-*O*-galloy-beta-D-glucose: Potential inhibitors of SARS-CoV-2 variants. Phys. Chem. Chem. Phys..

[36] Shen X., Zhang W., Peng C., Yan J., Chen P., Jiang C., Yuan Y., Chen D., Zhu W., Yao M. (2021). In vitro anti-bacterial activity and network pharmacology analysis of *Sanguisorba officinalis* L. against *Helicobacter pylori* infection. Chin. Med..

