# Supplementary material for: 1,3,6-Trigalloylglucose: A Novel Potent Anti-Helicobacter pylori Adhesion Agent Derived from Aqueous Extracts of Terminalia chebula Retz
Source: Molecules. 2024 Mar 5;29(5):1161. doi: 10.3390/molecules29051161 (PMC10935070; doi:10.3390/molecules29051161)
Supplement: Supplementary file 1 [file molecules-29-01161-s001.zip › molecules-2864401-supplementary.pdf]

Figure S1: the information of compound 3 by NMR analysis

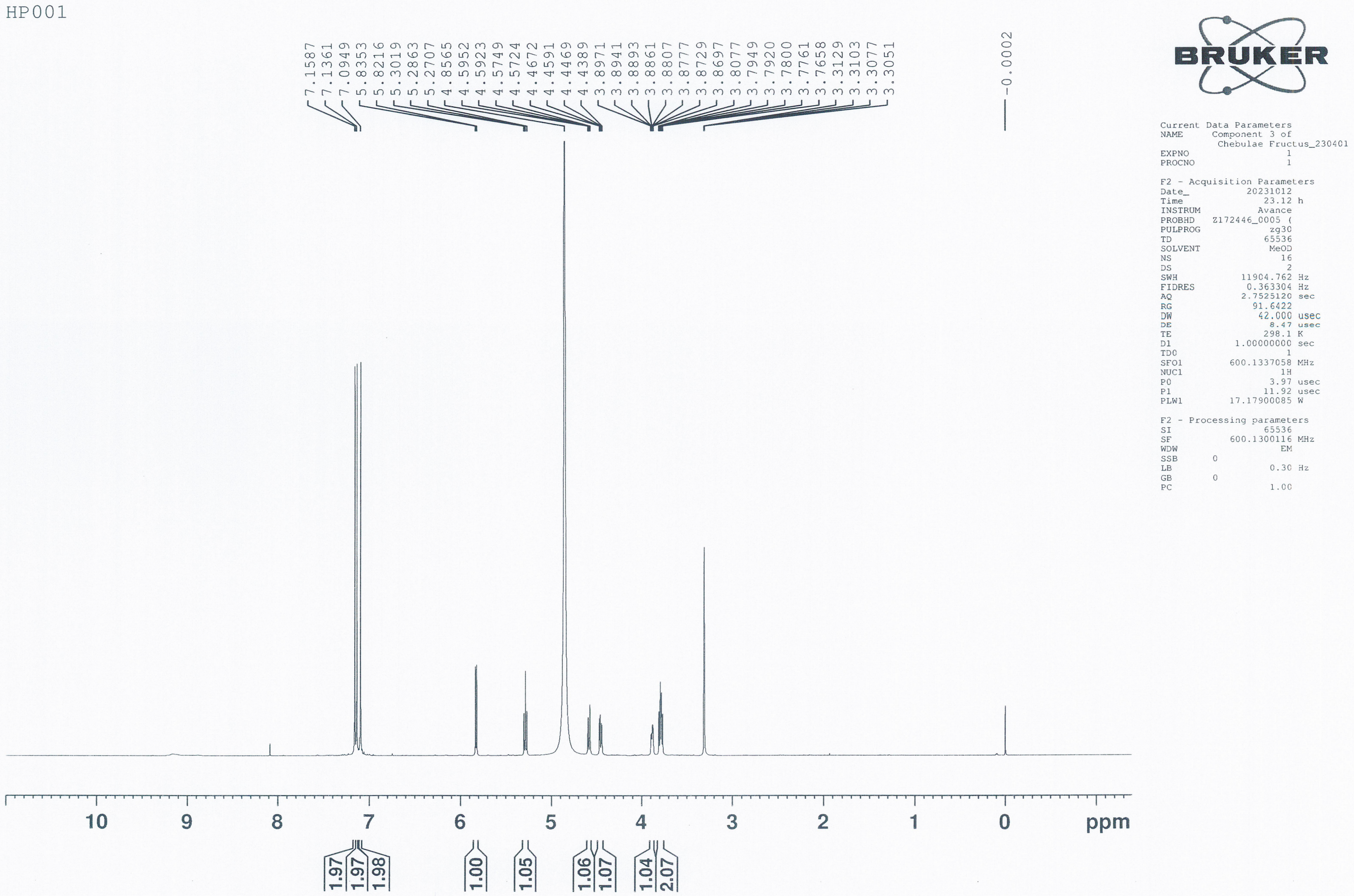

HP001

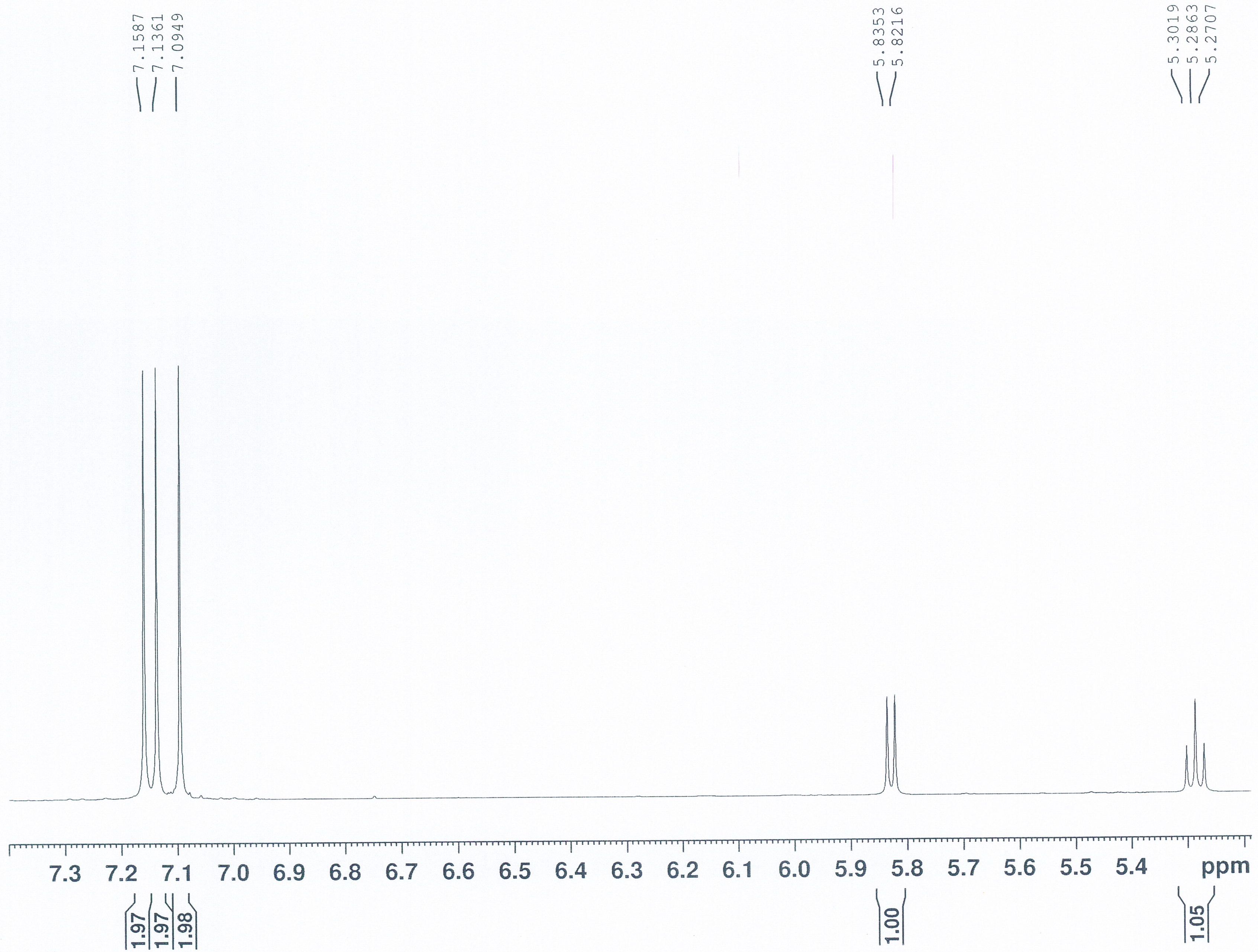

BRUKER

Current Data Parameters	
NAME	Component 3 of Chebulae Fructus_230401
EXPNO	1
PROCNO	1
F2 - Acquisition Parameters	
Date_	20231012
Time	23.12 h
INSTRUM	Avance
PROBHD	Z172446_0005 (
PULPROG	zg30
TD	65536
SOLVENT	MeOD
NS	16
DS	2
SWH	11904.762 Hz
FIDRES	0.363304 Hz
AQ	2.7525120 sec
RG	91.6422
DW	42.000 usec
DE	8.47 usec
TE	298.1 K
D1	1.00000000 sec
TD0	1
SFO1	600.1337058 MHz
NUC1	1H
P0	3.97 usec
P1	11.92 usec
PLW1	17.17900085 W
F2 - Processing parameters	
SI	65536
SF	600.1300116 MHz
WDW	EM
SSB	0
LB	0.30 Hz
GB	0
PC	1.00

HP001

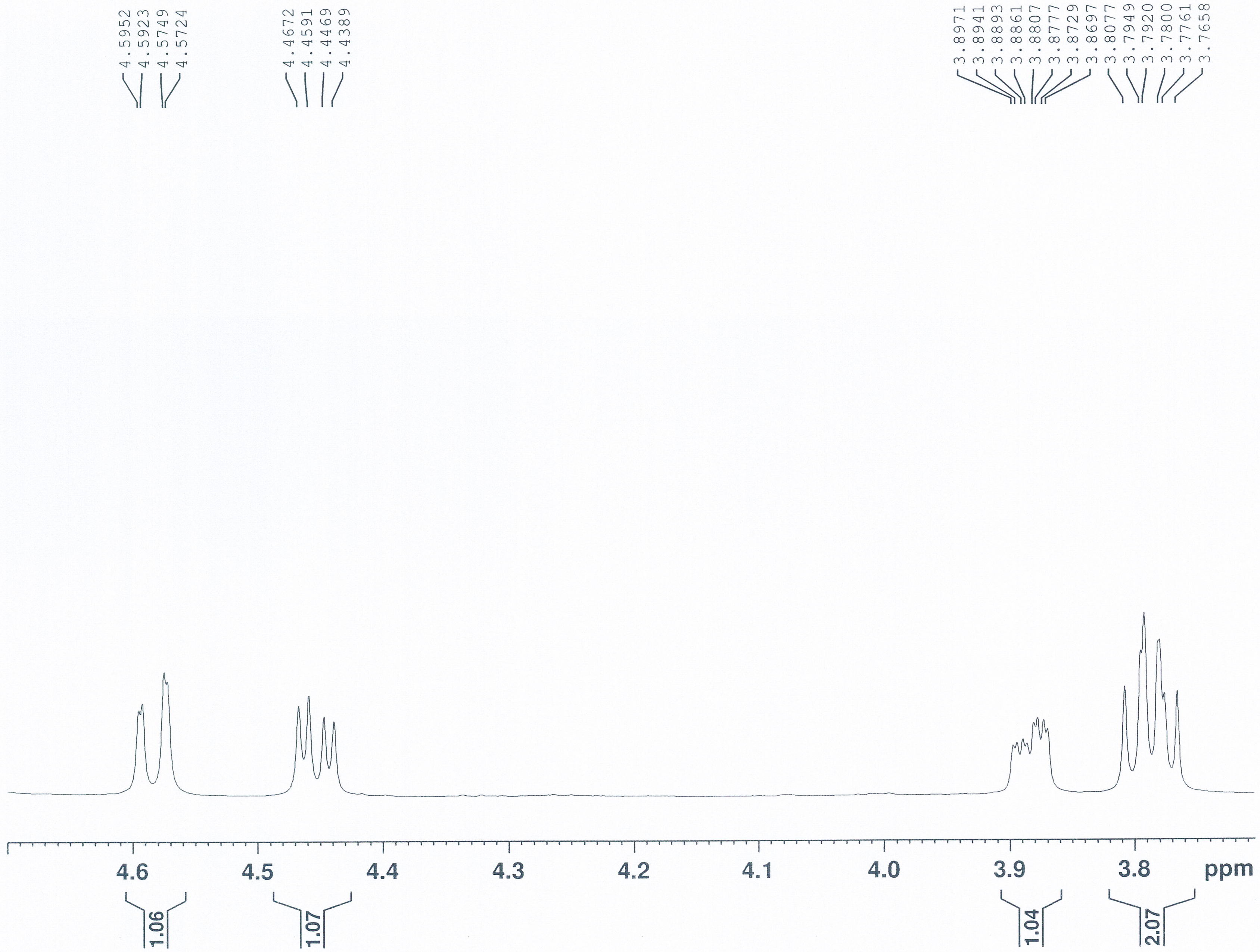

BRUKER

```
Current Data Parameters
NAME       Component 3 of
           Chebulae Fructus_230401
EXPNO                    1
PROCNO                   1

F2 - Acquisition Parameters
Date_             20231012
Time                 23.12 h
INSTRUM             Avance
PROBHD     Z172446_0005 (
PULPROG               zg30
TD                   65536
SOLVENT               MeOD
NS                      16
DS                       2
SWH              11904.762 Hz
FIDRES            0.363304 Hz
AQ               2.7525120 sec
RG                 91.6422
DW                  42.000 usec
DE                    8.47 usec
TE                   298.1 K
D1              1.00000000 sec
TD0                      1
SFO1          600.1337058 MHz
NUC1                    1H
P0                    3.97 usec
P1                   11.92 usec
PLW1           17.17900085 W

F2 - Processing parameters
SI                   65536
SF             600.1300116 MHz
WDW                     EM
SSB      0
LB                    0.30 Hz
GB       0
PC                    1.00
```

HP001

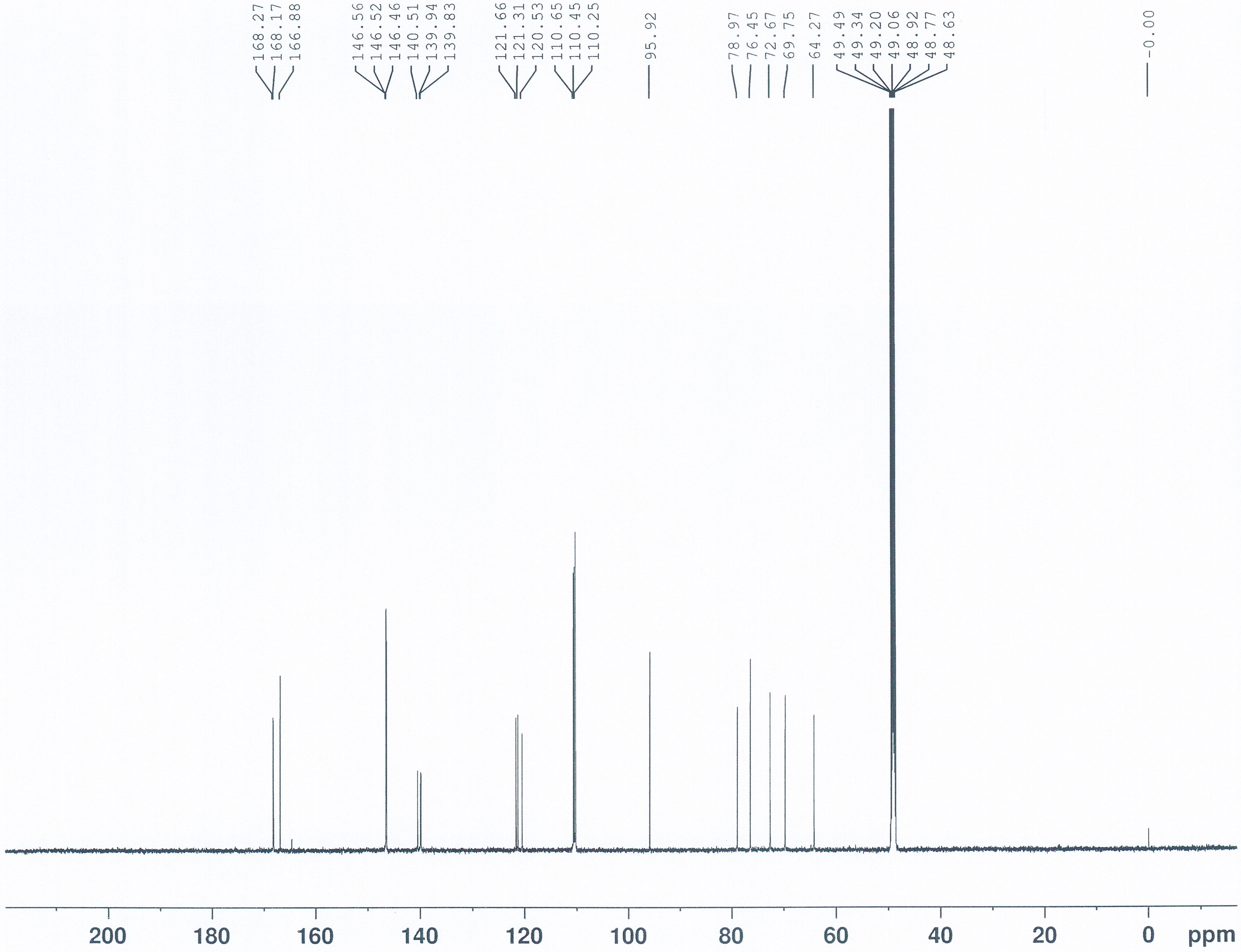

BRUKER

```
Current Data Parameters
NAME      Component 3 of
          Chebulae Fructus_230401
EXPNO                    2
PROCNO                   1

F2 - Acquisition Parameters
Date_             20231013
Time                  5.53 h
INSTRUM             Avance
PROBHD    Z172446_0005 (
PULPROG             zgpg30
TD                   65536
SOLVENT               MeOD
NS                    8000
DS                       4
SWH              35714.285 Hz
FIDRES            1.089913 Hz
AQ               0.9175040 sec
RG                     101
DW                  14.000 usec
DE                    6.50 usec
TE                   298.1 K
D1              2.00000000 sec
D11             0.03000000 sec
TD0                      1
SFO1           150.9178988 MHz
NUC1                   13C
P0                    3.93 usec
P1                   11.80 usec
PLW1           86.66300201 W
SFO2           600.1324005 MHz
NUC2                    1H
CPDPRG[2           waltz65
PCPD2                70.00 usec
PLW2           17.17900085 W
PLW12           0.49814001 W
PLW13           0.25055999 W

F2 - Processing parameters
SI                   32768
SF             150.9025931 MHz
WDW                     EM
SSB      0
LB                    1.00 Hz
GB       0
PC                    1.40
```

HP001

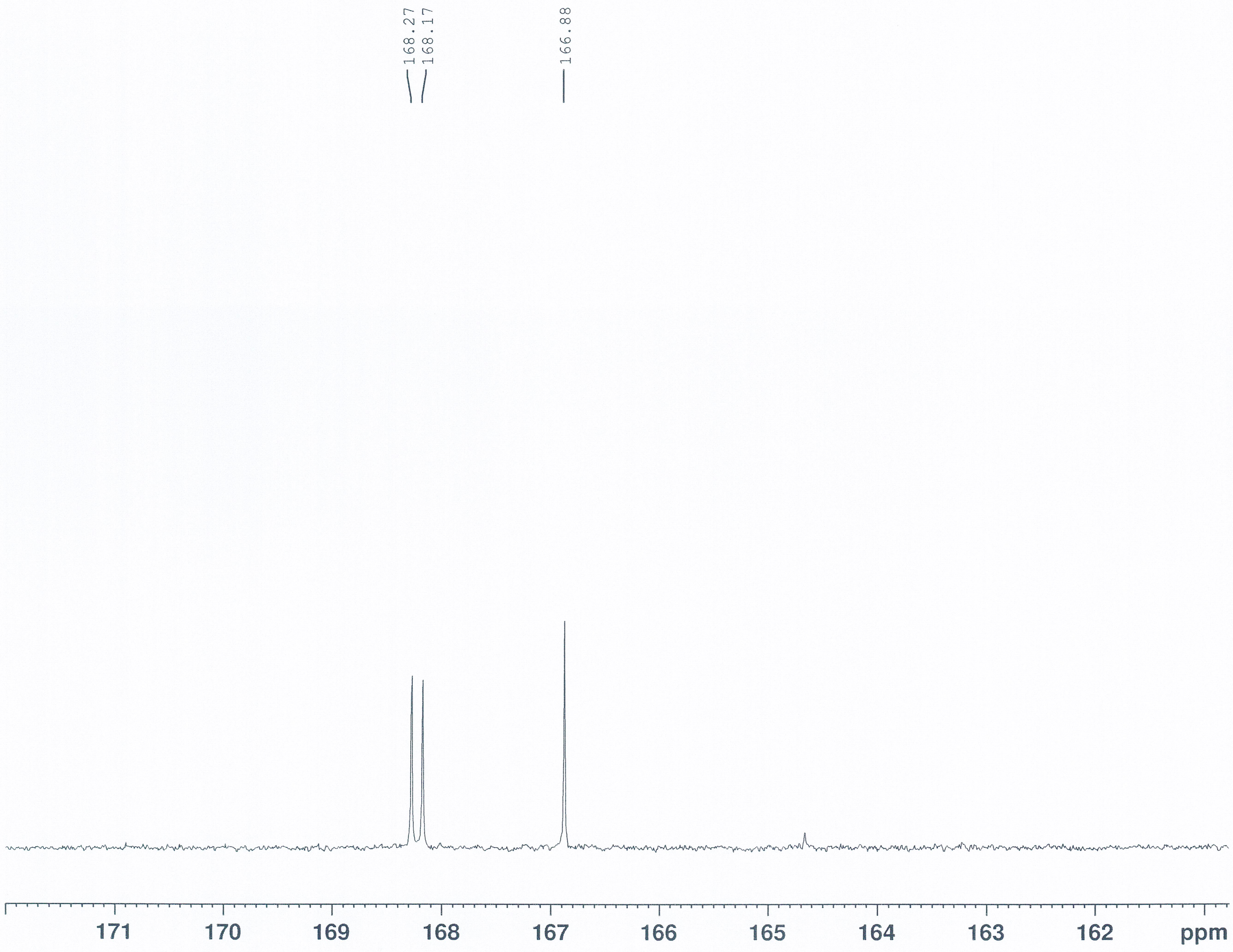

BRUKER

```
Current Data Parameters
NAME     Component 3 of
           Chebulae Fructus_230401
EXPNO                   2
PROCNO                  1

F2 - Acquisition Parameters
Date_            20231013
Time                 5.53 h
INSTRUM            Avance
PROBHD    Z172446_0005 (
PULPROG           zgpg30
TD                 65536
SOLVENT             MeOD
NS                  8000
DS                     4
SWH            35714.285 Hz
FIDRES          1.089913 Hz
AQ             0.9175040 sec
RG                   101
DW                14.000 usec
DE                  6.50 usec
TE                 298.1 K
D1            2.00000000 sec
D11           0.03000000 sec
TD0                    1
SFO1         150.9178988 MHz
NUC1                 13C
P0                  3.93 usec
P1                 11.80 usec
PLW1         86.66300201 W
SFO2         600.1324005 MHz
NUC2                  1H
CPDPRG[2          waltz65
PCPD2              70.00 usec
PLW2         17.17900085 W
PLW12         0.49814001 W
PLW13         0.25055999 W

F2 - Processing parameters
SI                 32768
SF           150.9025931 MHz
WDW                   EM
SSB        0
LB                  1.00 Hz
GB         0
PC                  1.40
```

HP001

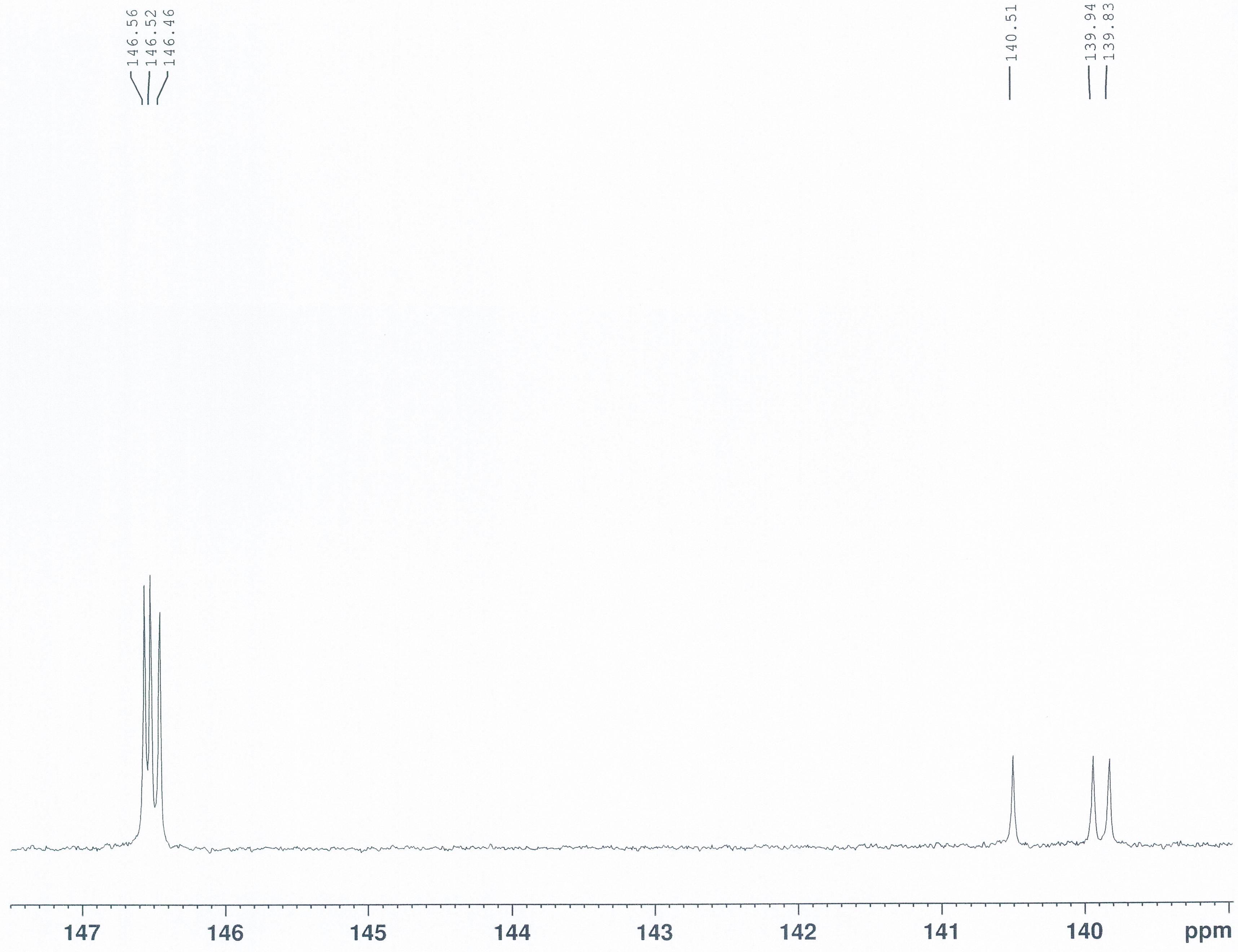

BRUKER

Current Data Parameters		
NAME	Component 3 of Chebulae Fructus_230401	
EXPNO	2	
PROCNO	1	
F2 - Acquisition Parameters		
Date_	20231013	
Time	5.53	h
INSTRUM	Avance	
PROBHD	Z172446_0005 (	
PULPROG	zgpg30	
TD	65536	
SOLVENT	MeOD	
NS	8000	
DS	4	
SWH	35714.285	Hz
FIDRES	1.089913	Hz
AQ	0.9175040	sec
RG	101	
DW	14.000	usec
DE	6.50	usec
TE	298.1	K
D1	2.00000000	sec
D11	0.03000000	sec
TD0	1	
SFO1	150.9178988	MHz
NUC1	13C	
P0	3.93	usec
P1	11.80	usec
PLW1	86.66300201	W
SFO2	600.1324005	MHz
NUC2	1H	
CPDPRG[2	waltz65	
PCPD2	70.00	usec
PLW2	17.17900085	W
PLW12	0.49814001	W
PLW13	0.25055999	W
F2 - Processing parameters		
SI	32768	
SF	150.9025931	MHz
WDW	EM	
SSB	0	
LB	1.00	Hz
GB	0	
PC	1.40	

HP001

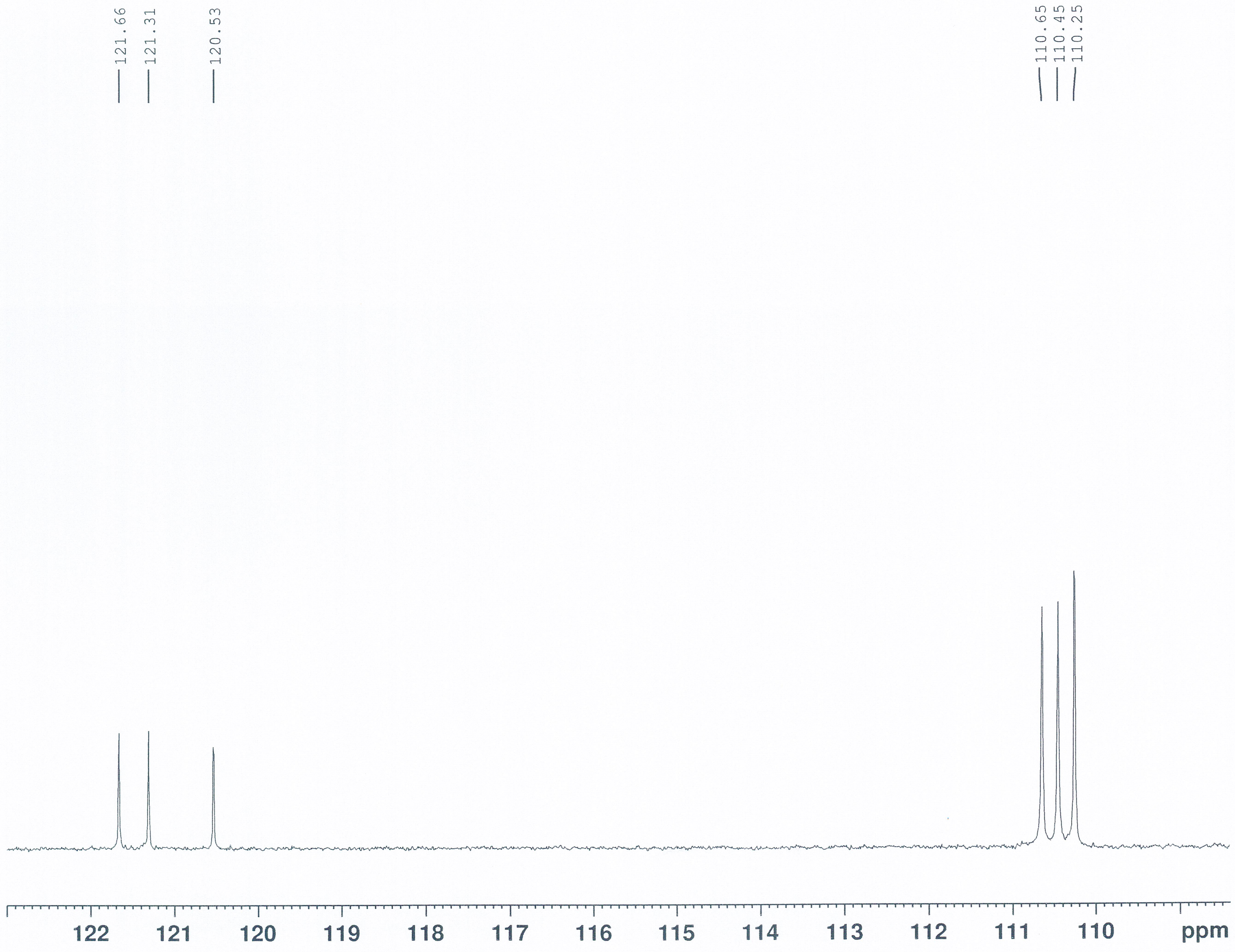

BRUKER

Current Data Parameters
NAME Component 3 of Chebulae Fructus_230401
EXPNO 2
PROCNO 1

F2 - Acquisition Parameters

Date_	20231013	
Time	5.53	h
INSTRUM	Avance	
PROBHD	Z172446_0005 (	
PULPROG	zgpg30	
TD	65536	
SOLVENT	MeOD	
NS	8000	
DS	4	
SWH	35714.285	Hz
FIDRES	1.089913	Hz
AQ	0.9175040	sec
RG	101	
DW	14.000	usec
DE	6.50	usec
TE	298.1	K
D1	2.00000000	sec
D11	0.03000000	sec
TD0	1	
SFO1	150.9178988	MHz
NUC1	13C	
P0	3.93	usec
P1	11.80	usec
PLW1	86.66300201	W
SFO2	600.1324005	MHz
NUC2	1H	
CPDPRG[2	waltz65	
PCPD2	70.00	usec
PLW2	17.17900085	W
PLW12	0.49814001	W
PLW13	0.25055999	W

F2 - Processing parameters

SI	32768	
SF	150.9025931	MHz
WDW	EM	
SSB	0	
LB	1.00	Hz
GB	0	
PC	1.40	

HP001

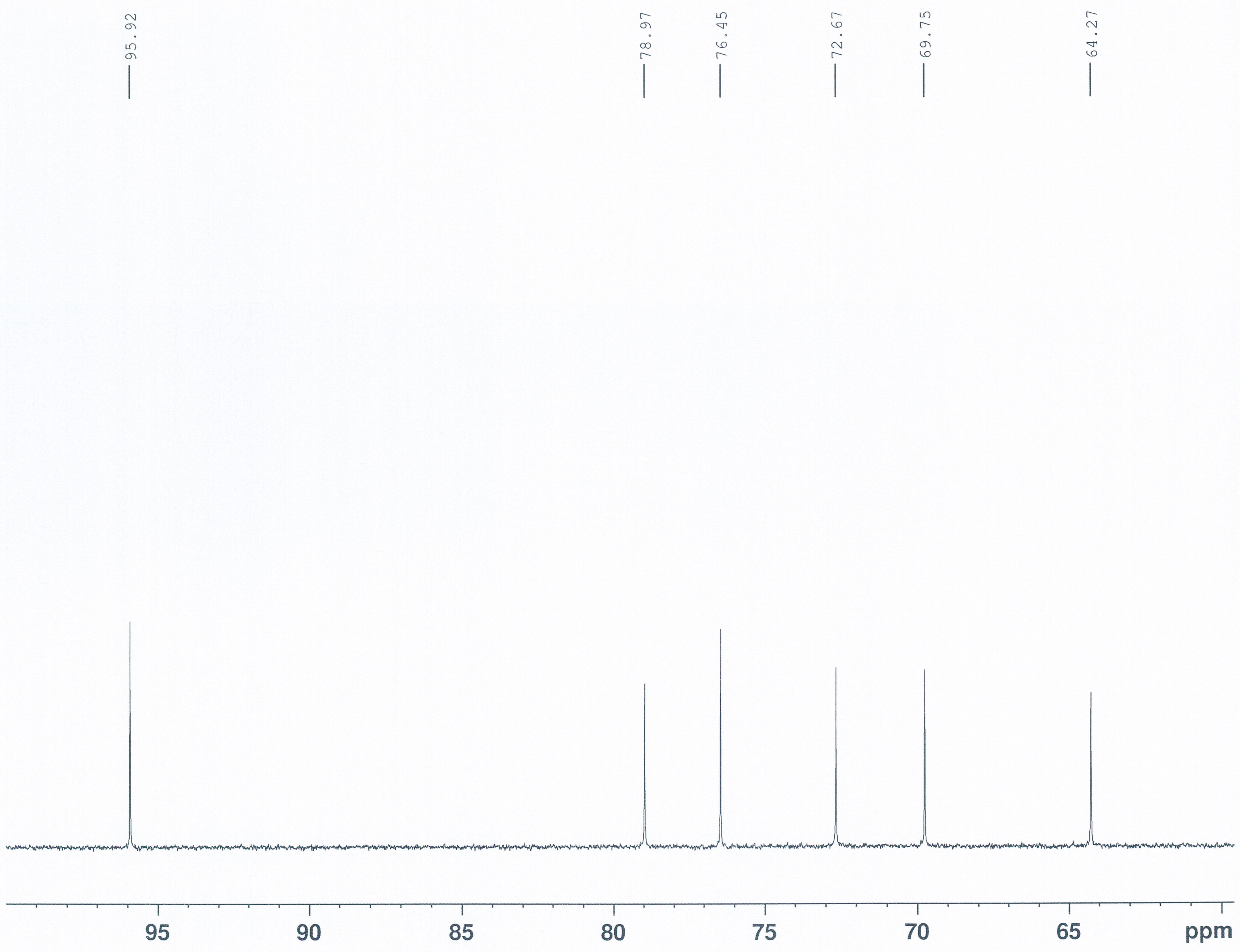

BRUKER

```
Current Data Parameters
NAME       Component 3 of
             Chebulae Fructus_230401
EXPNO                  2
PROCNO                 1

F2 - Acquisition Parameters
Date_           20231013
Time                5.53 h
INSTRUM           Avance
PROBHD    Z172446_0005 (
PULPROG           zgpg30
TD                 65536
SOLVENT             MeOD
NS                  8000
DS                     4
SWH            35714.285 Hz
FIDRES          1.089913 Hz
AQ             0.9175040 sec
RG                   101
DW                14.000 usec
DE                  6.50 usec
TE                 298.1 K
D1            2.00000000 sec
D11           0.03000000 sec
TD0                    1
SFO1         150.9178988 MHz
NUC1                 13C
P0                  3.93 usec
P1                 11.80 usec
PLW1         86.66300201 W
SFO2         600.1324005 MHz
NUC2                  1H
CPDPRG[2         waltz65
PCPD2              70.00 usec
PLW2         17.17900085 W
PLW12         0.49814001 W
PLW13         0.25055999 W

F2 - Processing parameters
SI                 32768
SF           150.9025931 MHz
WDW                   EM
SSB      0
LB                  1.00 Hz
GB       0
PC                  1.40
```

HP001

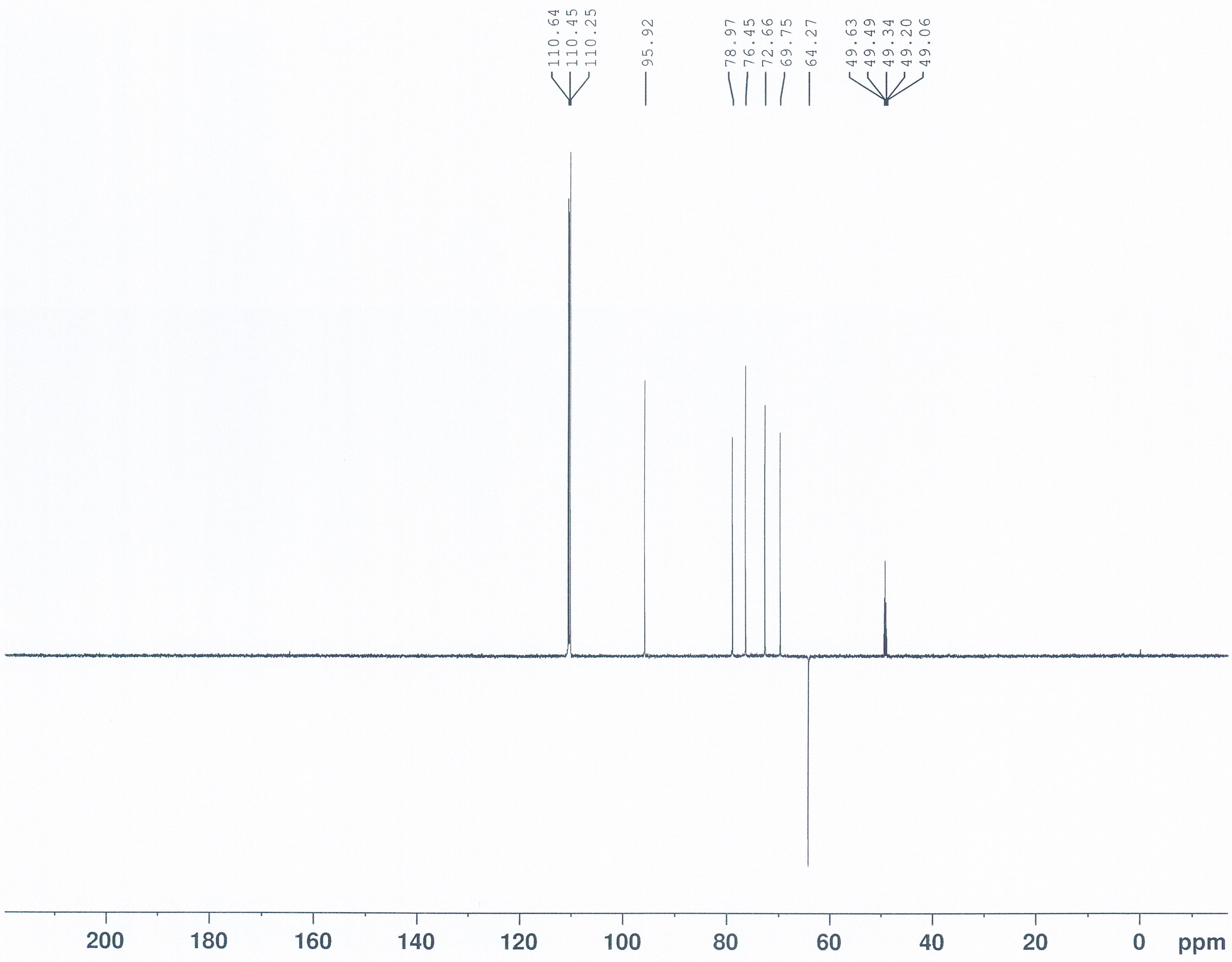

BRUKER

Current Data Parameters

NAME	Component 3 of Chebulae Fructus_230401
EXPNO	3
PROCNO	1

F2 - Acquisition Parameters

Date_	20231013
Time	9.12 h
INSTRUM	Avance
PROBHD	Z172446_0005 (
PULPROG	deptsp135
TD	65536
SOLVENT	MeOD
NS	4000
DS	8
SWH	35714.285 Hz
FIDRES	1.089913 Hz
AQ	0.9175040 sec
RG	101
DW	14.000 usec
DE	6.50 usec
TE	298.2 K
CNST2	145.0000000
D1	2.00000000 sec
D2	0.00344828 sec
D12	0.00002000 sec
TD0	1
SFO1	150.9178988 MHz
NUC1	13C
P1	11.80 usec
P13	2000.00 usec
PLW0	0 W
PLW1	86.66300201 W
SPNAM[5]	Crp60comp.4
SPOAL5	0.500
SPOFFS5	0 Hz
SPW5	18.43700027 W
SFO2	600.1324005 MHz
NUC2	1H
CPDPRG[2	waltz65
P3	11.92 usec
P4	23.84 usec
PCPD2	70.00 usec
PLW2	17.17900085 W
PLW12	0.49814001 W

F2 - Processing parameters

SI	32768
SF	150.9025931 MHz
WDW	EM
SSB	0
LB	1.00 Hz
GB	0
PC	1.40

HP001

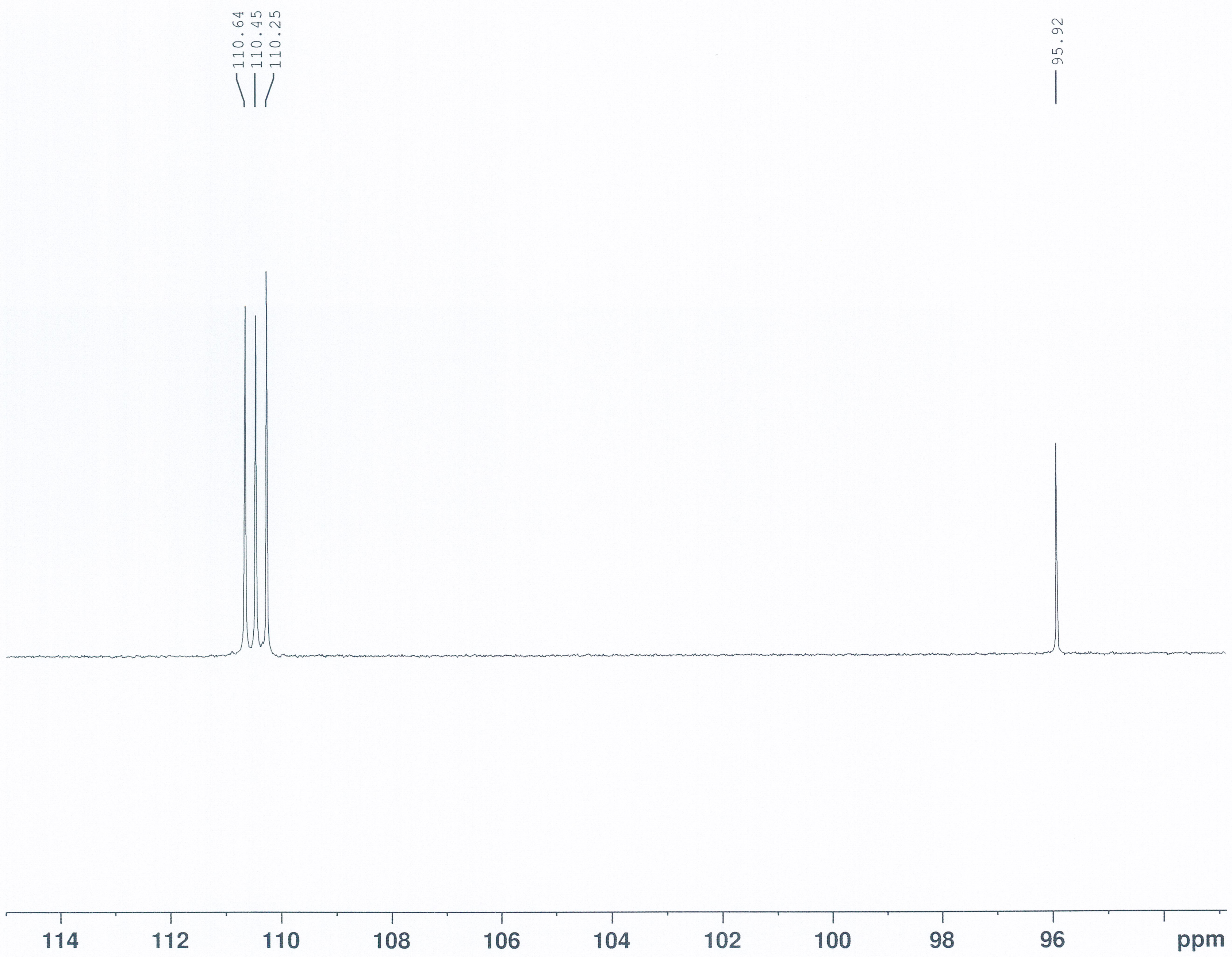

Current Data Parameters

NAME	Component 3 of Chebulae Fructus_230401
EXPNO	3
PROCNO	1

F2 - Acquisition Parameters

Date_	20231013
Time	9.12 h
INSTRUM	Avance
PROBHD	Z172446_0005 (
PULPROG	deptsp135
TD	65536
SOLVENT	MeOD
NS	4000
DS	8
SWH	35714.285 Hz
FIDRES	1.089913 Hz
AQ	0.9175040 sec
RG	101
DW	14.000 usec
DE	6.50 usec
TE	298.2 K
CNST2	145.0000000
D1	2.00000000 sec
D2	0.00344828 sec
D12	0.00002000 sec
TD0	1
SFO1	150.9178988 MHz
NUC1	13C
P1	11.80 usec
P13	2000.00 usec
PLW0	0 W
PLW1	86.66300201 W
SPNAM[5]	Crp60comp.4
SPOAL5	0.500
SPOFFS5	0 Hz
SPW5	18.43700027 W
SFO2	600.1324005 MHz
NUC2	1H
CPDPRG[2	waltz65
P3	11.92 usec
P4	23.84 usec
PCPD2	70.00 usec
PLW2	17.17900085 W
PLW12	0.49814001 W

F2 - Processing parameters

SI	32768
SF	150.9025931 MHz
WDW	EM
SSB	0
LB	1.00 Hz
GB	0
PC	1.40

HP001

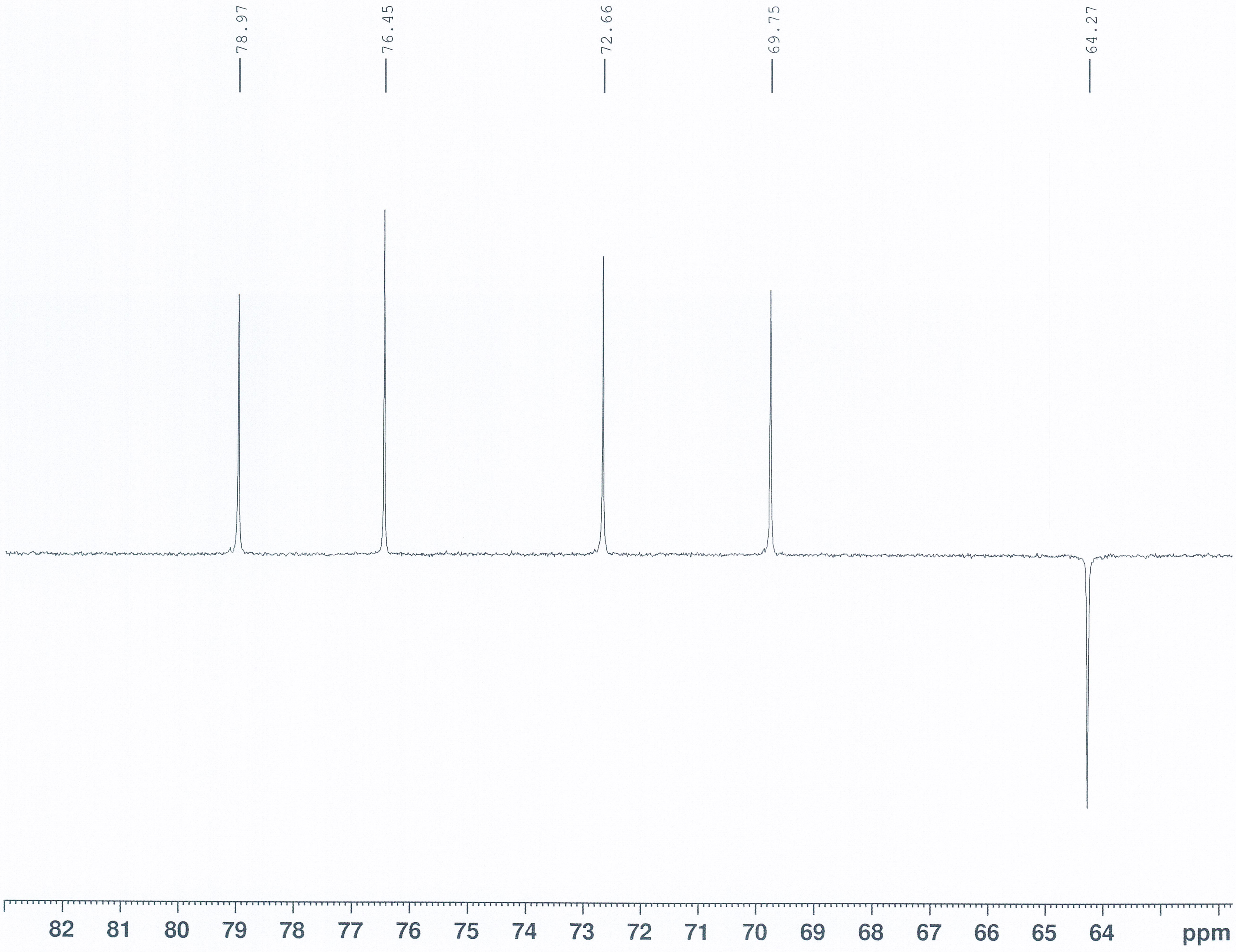

BRUKER

Current Data Parameters
NAME Component 3 of Chebulae Fructus_230401
EXPNO 3
PROCNO 1

F2 - Acquisition Parameters

Date_	20231013
Time	9.12 h
INSTRUM	Avance
PROBHD	Z172446_0005 (
PULPROG	deptsp135
TD	65536
SOLVENT	MeOD
NS	4000
DS	8
SWH	35714.285 Hz
FIDRES	1.089913 Hz
AQ	0.9175040 sec
RG	101
DW	14.000 usec
DE	6.50 usec
TE	298.2 K
CNST2	145.0000000
D1	2.00000000 sec
D2	0.00344828 sec
D12	0.00002000 sec
TD0	1
SFO1	150.9178988 MHz
NUC1	13C
P1	11.80 usec
P13	2000.00 usec
PLW0	0 W
PLW1	86.66300201 W
SPNAM[5]	Crp60comp.4
SPOAL5	0.500
SPOFFS5	0 Hz
SPW5	18.43700027 W
SFO2	600.1324005 MHz
NUC2	1H
CPDPRG[2	waltz65
P3	11.92 usec
P4	23.84 usec
PCPD2	70.00 usec
PLW2	17.17900085 W
PLW12	0.49814001 W

F2 - Processing parameters

SI	32768
SF	150.9025931 MHz
WDW	EM
SSB	0
LB	1.00 Hz
GB	0
PC	1.40

HP001

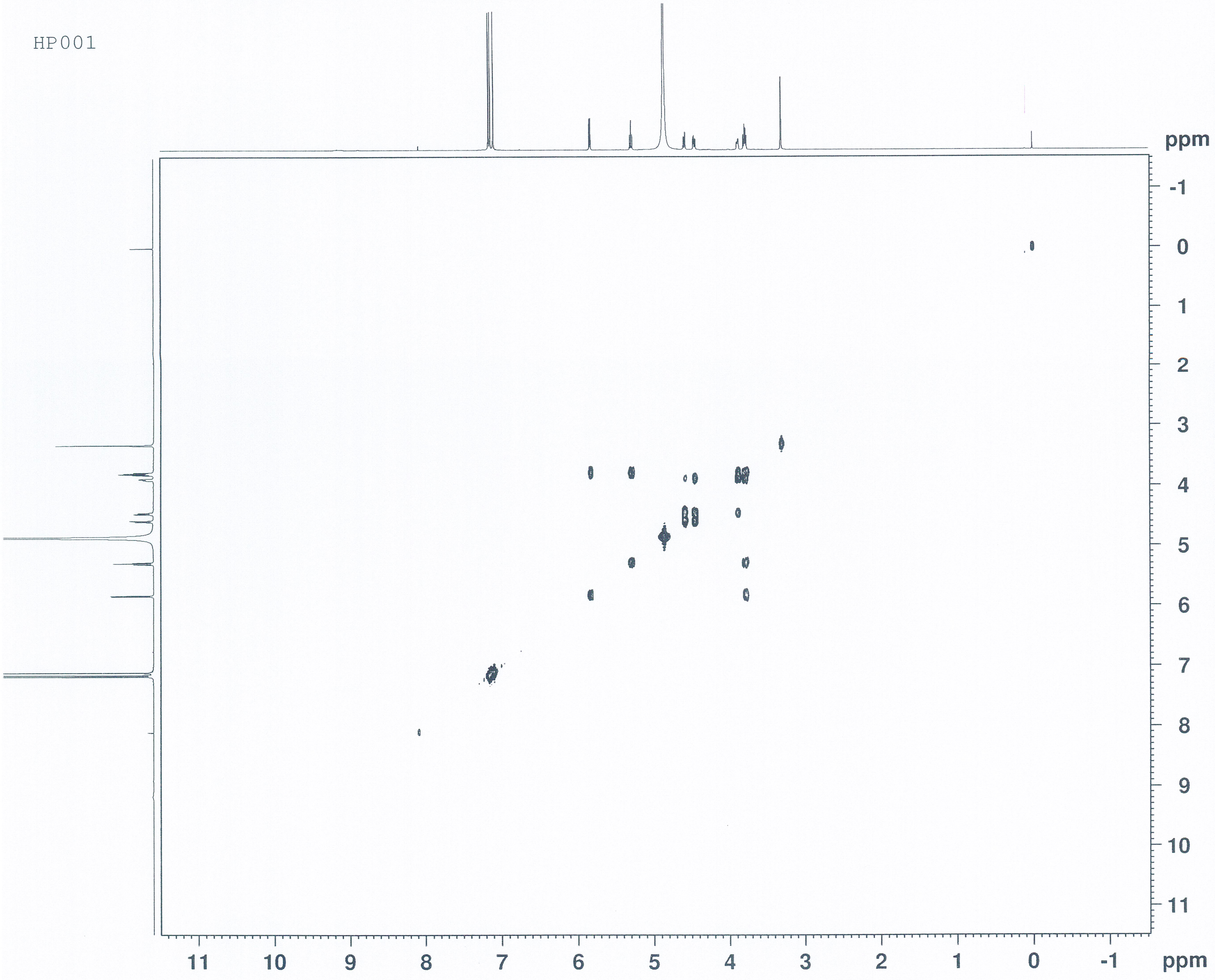

Current Data Parameters

NAME	Component 3 of Chebulae Fructus_230401
EXPNO	4
PROCNO	1

F2 - Acquisition Parameters

Date_	20231013
Time	9.59 h
INSTRUM	Avance
PROBHD	Z172446_0005 (
PULPROG	cosygpppqf
TD	2048
SOLVENT	MeOD
NS	10
DS	16
SWH	7812.500 Hz
FIDRES	7.629395 Hz
AQ	0.1310720 sec
RG	101
DW	64.000 usec
DE	6.50 usec
TE	298.1 K
D0	0.00000300 sec
D1	2.00000000 sec
D11	0.03000000 sec
D12	0.00002000 sec
D13	0.00000400 sec
D16	0.00020000 sec
IN0	0.00012800 sec
TDav	1
SFO1	600.1330006 MHz
NUC1	1H
P0	11.92 usec
P1	11.92 usec
P17	2500.00 usec
PLW1	17.17900085 W
PLW10	3.90540004 W
GPNAM[1]	SMSQ10.100
GPZ1	10.00 %
P16	1000.00 usec

F1 - Acquisition parameters

TD	128
SFO1	600.133 MHz
FIDRES	122.070313 Hz
SW	13.018 ppm
FnMODE	QF

F2 - Processing parameters

SI	1024
SF	600.1300116 MHz
WDW	QSINE
SSB	0
LB	0 Hz
GB	0
PC	1.40

F1 - Processing parameters

SI	1024
MC2	QF
SF	600.1300116 MHz
WDW	QSINE
SSB	0
LB	0 Hz
GB	0

HP001

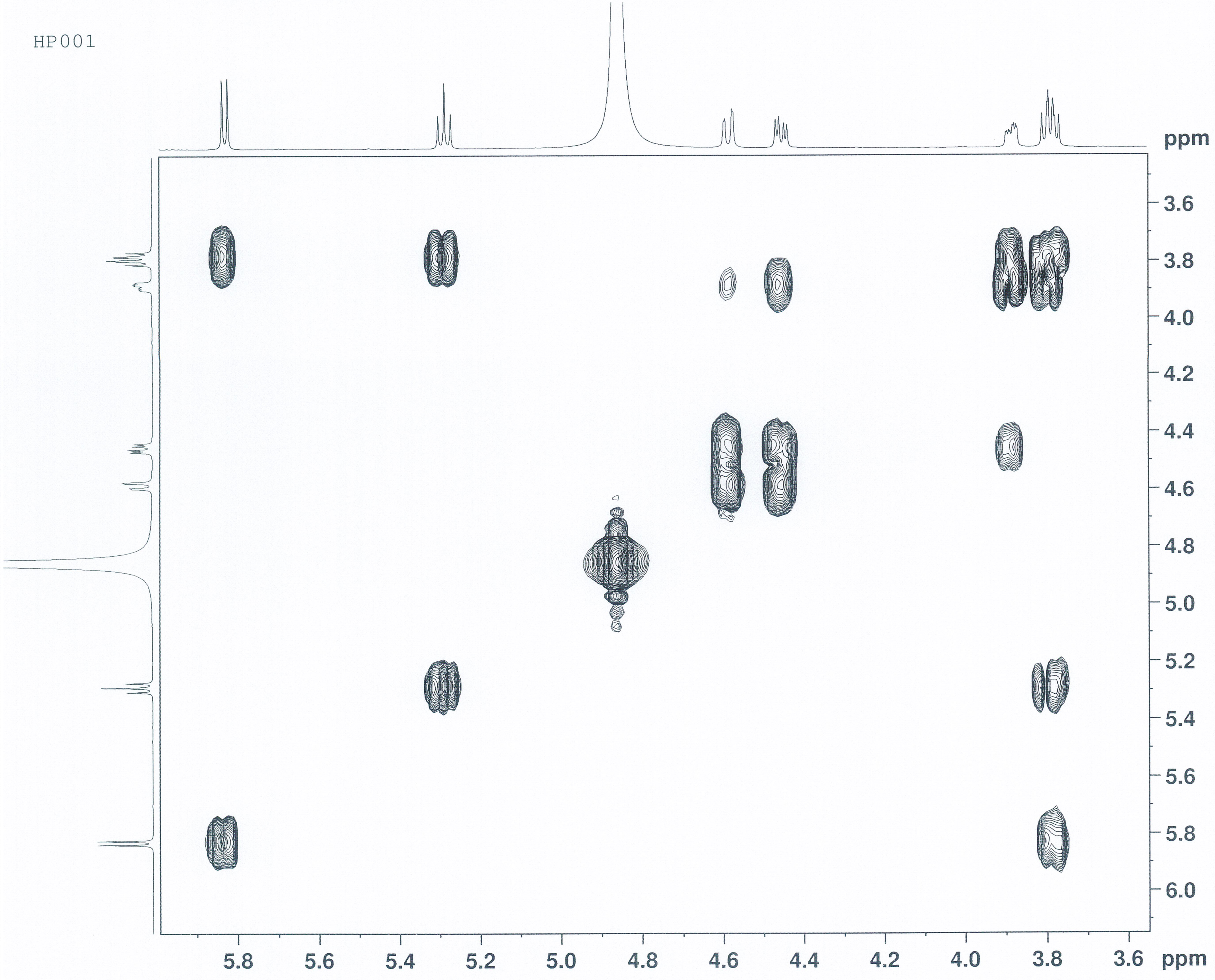

Current Data Parameters
NAME Component 3 of Chebulae Fructus_230401
EXPNO 4
PROCNO 1

F2 - Acquisition Parameters
Date_ 20231013
Time 9.59 h
INSTRUM Avance
PROBHD Z172446_0005 (
PULPROG cosygpppqf
TD 2048
SOLVENT MeOD
NS 10
DS 16
SWH 7812.500 Hz
FIDRES 7.629395 Hz
AQ 0.1310720 sec
RG 101
DW 64.000 usec
DE 6.50 usec
TE 298.1 K
D0 0.00000300 sec
D1 2.00000000 sec
D11 0.03000000 sec
D12 0.00002000 sec
D13 0.00000400 sec
D16 0.00020000 sec
IN0 0.00012800 sec
TDav 1
SFO1 600.1330006 MHz
NUC1 1H
P0 11.92 usec
P1 11.92 usec
P17 2500.00 usec
PLW1 17.17900085 W
PLW10 3.90540004 W
GPNAM[1] SMSQ10.100
GPZ1 10.00 %
P16 1000.00 usec

F1 - Acquisition parameters
TD 128
SFO1 600.133 MHz
FIDRES 122.070313 Hz
SW 13.018 ppm
FnMODE QF

F2 - Processing parameters
SI 1024
SF 600.1300116 MHz
WDW QSINE
SSB 0
LB 0 Hz
GB 0
PC 1.40

F1 - Processing parameters
SI 1024
MC2 QF
SF 600.1300116 MHz
WDW QSINE
SSB 0
LB 0 Hz
GB 0

HP001

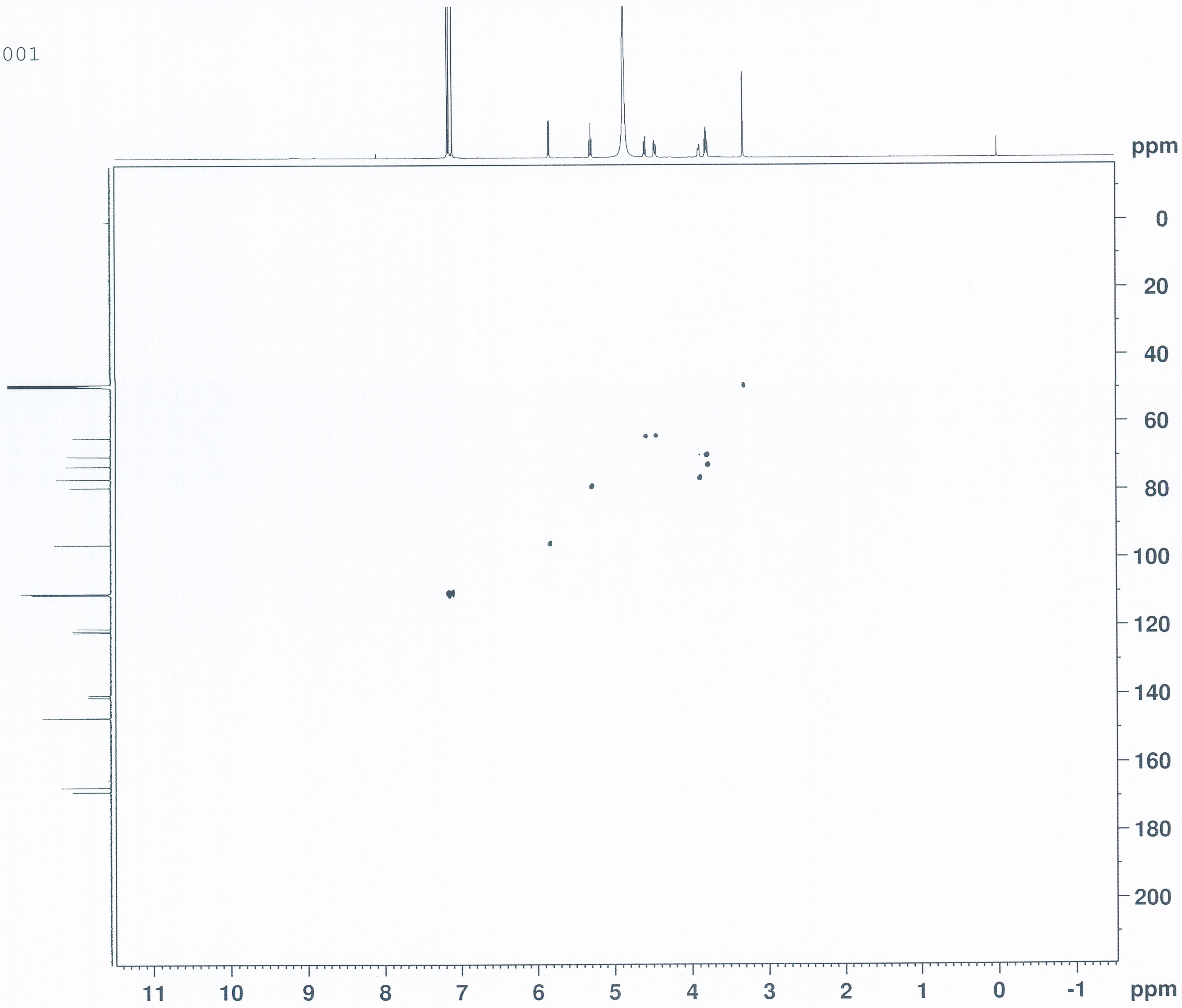

BRUKER

```
Current Data Parameters
NAME       Component 3 of
            Chebulae Fructus_230401
EXPNO                    5
PROCNO                   1

F2 - Acquisition Parameters
Date_             20231013
Time                 11.34 h
INSTRUM             Avance
PROBHD     Z172446_0005 (
PULPROG   hsqcedetgpsisp2.3
TD                    2048
SOLVENT               MeOD
NS                      10
DS                      32
SWH               7812.500 Hz
FIDRES            7.629395 Hz
AQ               0.1310720 sec
RG                     101
DW                  64.000 usec
DE                    6.50 usec
TE                   298.2 K
CNST2          145.0000000
CNST17          -0.5000000
D0              0.00000300 sec
D1              2.00000000 sec
D4              0.00172414 sec
D11             0.03000000 sec
D16             0.00020000 sec
D21             0.00360000 sec
D24             0.00089000 sec
IN0             0.00001404 sec
TDav                     1
SFO1           600.1330006 MHz
NUC1                    1H
P1                   11.92 usec
P2                   23.84 usec
P28                1000.00 usec
PLW1           17.17900085 W
SFO2           150.9178988 MHz
NUC2                   13C
CPDPRG[2 bi_p5m4sp_4sp.2
P3                   11.80 usec
P14                 500.00 usec
P24                2000.00 usec
P31                1730.00 usec
P63                1500.00 usec
PLW0       0 W
PLW2           86.66300201 W
PLW12           3.35190010 W
P16                1000.00 usec
P19                 600.00 usec

F1 - Acquisition parameters
TD                     256
SFO1              150.9179 MHz
FIDRES          278.272491 Hz
SW                 236.015 ppm
FnMODE      Echo-Antiecho

F2 - Processing parameters
SI                    1024
SF             600.1300116 MHz
WDW                  QSINE
SSB                      2
LB         0 Hz
GB         0
PC                    1.40

F1 - Processing parameters
SI                    1024
MC2          echo-antiecho
SF             150.9025931 MHz
WDW                  QSINE
SSB                      2
LB         0 Hz
GB         0
```

HP001

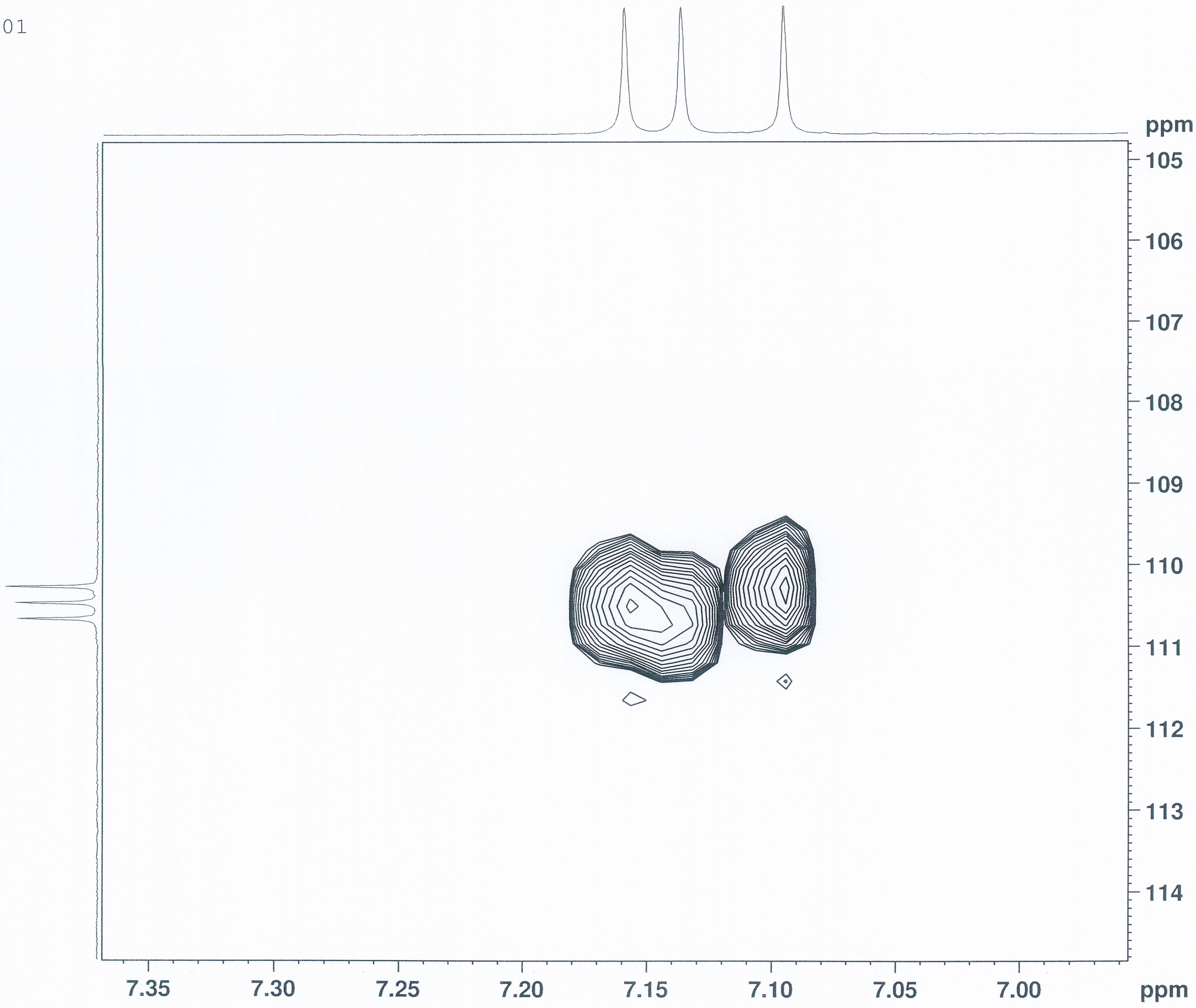

Current Data Parameters
NAME Component 3 of Chebulae Fructus_230401
EXPNO 5
PROCNO 1

F2 - Acquisition Parameters
Date_ 20231013
Time 11.34 h
INSTRUM Avance
PROBHD Z172446_0005 (
PULPROG hsqcedetgpsisp2.3
TD 2048
SOLVENT MeOD
NS 10
DS 32
SWH 7812.500 Hz
FIDRES 7.629395 Hz
AQ 0.1310720 sec
RG 101
DW 64.000 usec
DE 6.50 usec
TE 298.2 K
CNST2 145.0000000
CNST17 -0.5000000
D0 0.00000300 sec
D1 2.00000000 sec
D4 0.00172414 sec
D11 0.03000000 sec
D16 0.00020000 sec
D21 0.00360000 sec
D24 0.00089000 sec
IN0 0.00001404 sec
TDav 1
SFO1 600.1330006 MHz
NUC1 1H
P1 11.92 usec
P2 23.84 usec
P28 1000.00 usec
PLW1 17.17900085 W
SFO2 150.9178988 MHz
NUC2 13C
CPDPRG[2 bi_p5m4sp_4sp.2
P3 11.80 usec
P14 500.00 usec
P24 2000.00 usec
P31 1730.00 usec
P63 1500.00 usec
PLW0 0 W
PLW2 86.66300201 W
PLW12 3.35190010 W
P16 1000.00 usec
P19 600.00 usec

F1 - Acquisition parameters
TD 256
SFO1 150.9179 MHz
FIDRES 278.272491 Hz
SW 236.015 ppm
FnMODE Echo-Antiecho

F2 - Processing parameters
SI 1024
SF 600.1300116 MHz
WDW QSINE
SSB 2
LB 0 Hz
GB 0
PC 1.40

F1 - Processing parameters
SI 1024
MC2 echo-antiecho
SF 150.9025931 MHz
WDW QSINE
SSB 2
LB 0 Hz
GB 0

HP001

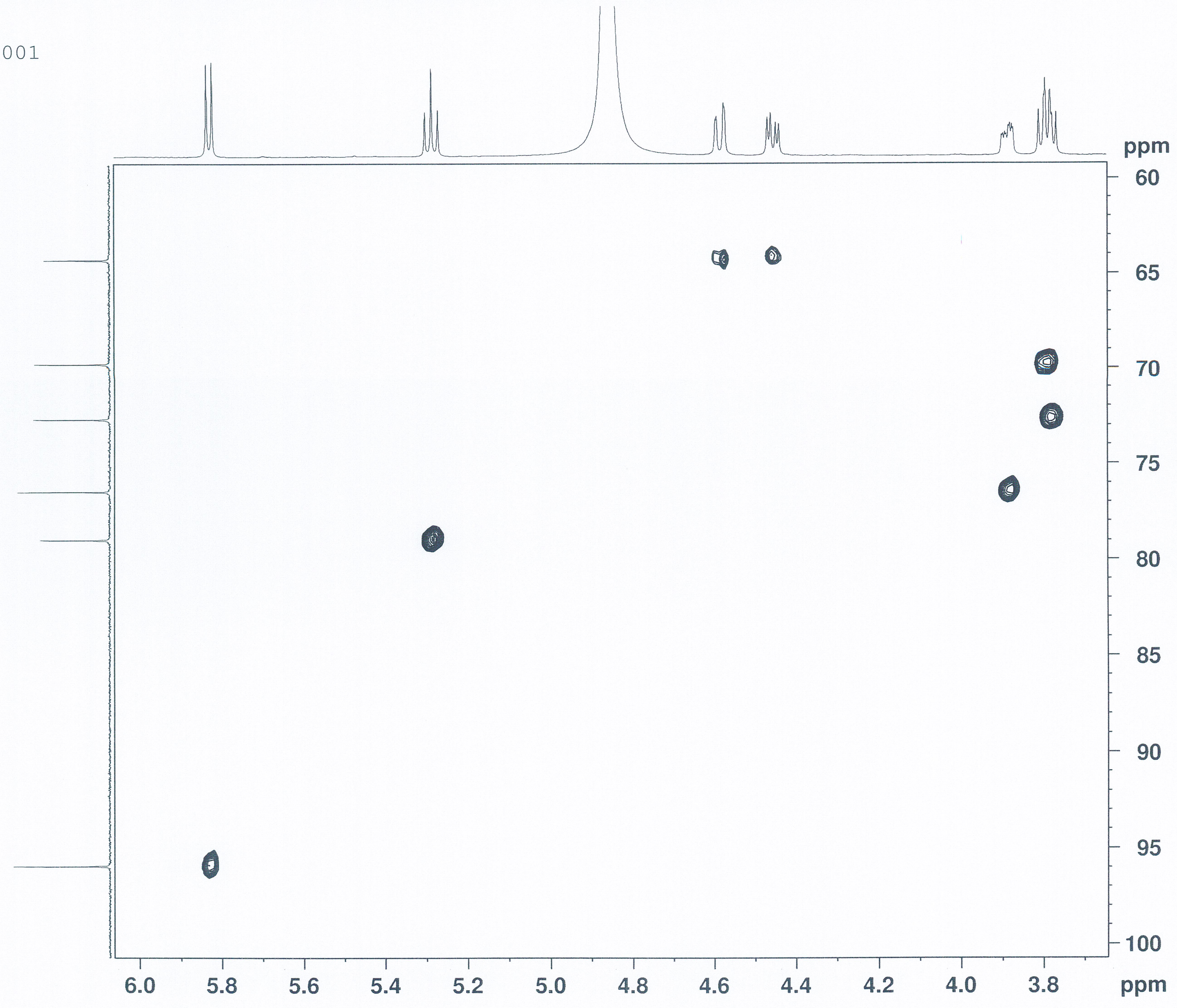

BRUKER

```
Current Data Parameters
NAME      Component 3 of
          Chebulae Fructus_230401
EXPNO                 5
PROCNO                1

F2 - Acquisition Parameters
Date_          20231013
Time              11.34 h
INSTRUM          Avance
PROBHD   Z172446_0005 (
PULPROG  hsqcedetgpsisp2.3
TD                 2048
SOLVENT            MeOD
NS                   10
DS                   32
SWH            7812.500 Hz
FIDRES         7.629395 Hz
AQ            0.1310720 sec
RG                  101
DW               64.000 usec
DE                 6.50 usec
TE                298.2 K
CNST2       145.0000000
CNST17       -0.5000000
D0           0.00000300 sec
D1           2.00000000 sec
D4           0.00172414 sec
D11          0.03000000 sec
D16          0.00020000 sec
D21          0.00360000 sec
D24          0.00089000 sec
IN0          0.00001404 sec
TDav                  1
SFO1        600.1330006 MHz
NUC1                 1H
P1                11.92 usec
P2                23.84 usec
P28             1000.00 usec
PLW1        17.17900085 W
SFO2        150.9178988 MHz
NUC2                13C
CPDPRG[2  bi_p5m4sp_4sp.2
P3                11.80 usec
P14              500.00 usec
P24             2000.00 usec
P31             1730.00 usec
P63             1500.00 usec
PLW0      0 W
PLW2        86.66300201 W
PLW12        3.35190010 W
P16             1000.00 usec
P19              600.00 usec

F1 - Acquisition parameters
TD                  256
SFO1           150.9179 MHz
FIDRES       278.272491 Hz
SW              236.015 ppm
FnMODE     Echo-Antiecho

F2 - Processing parameters
SI                 1024
SF          600.1300116 MHz
WDW               QSINE
SSB                   2
LB        0 Hz
GB        0
PC                 1.40

F1 - Processing parameters
SI                 1024
MC2         echo-antiecho
SF          150.9025931 MHz
WDW               QSINE
SSB                   2
LB        0 Hz
GB        0
```

HP001

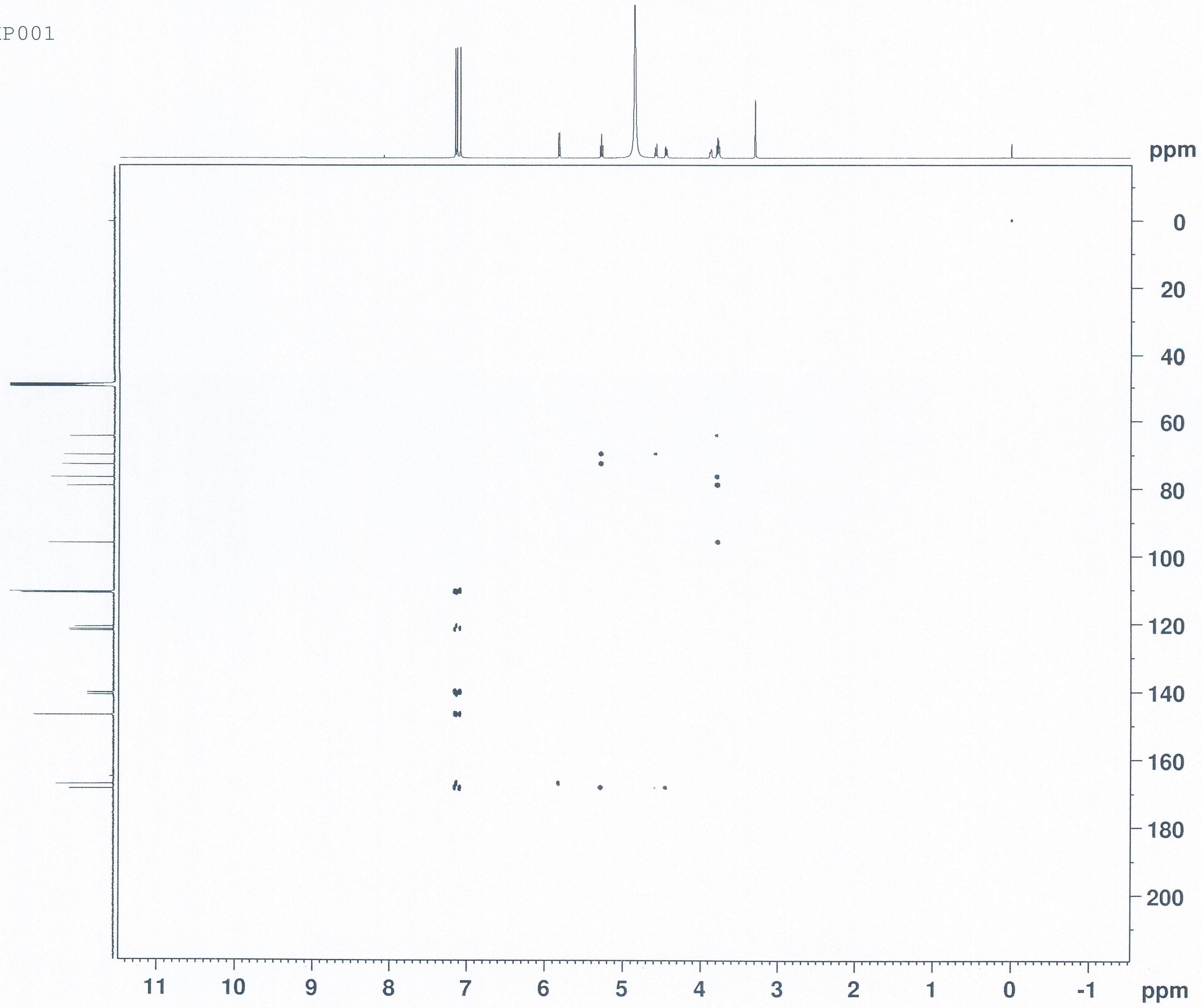

Current Data Parameters
NAME Component 3 of
Chebulae Fructus_230401
EXPNO 6
PROCNO 1

F2 - Acquisition Parameters
Date_ 20231013
Time 20.35 h
INSTRUM Avance
PROBHD Z172446_0005 (
PULPROG hmbcetgpl3nd
TD 4096
SOLVENT MeOD
NS 54
DS 16
SWH 7812.500 Hz
FIDRES 3.814697 Hz
AQ 0.2621440 sec
RG 101
DW 64.000 usec
DE 6.50 usec
TE 298.2 K
CNST6 120.0000000
CNST7 170.0000000
CNST13 8.0000000
D0 0.00000300 sec
D1 2.00000000 sec
D6 0.06250000 sec
D16 0.00020000 sec
IN0 0.00001404 sec
TDav 1
SFO1 600.1330006 MHz
NUC1 1H
P1 11.92 usec
P2 23.84 usec
PLW1 17.17900085 W
SFO2 150.9178988 MHz
NUC2 13C
P3 11.80 usec
P24 2000.00 usec
PLW2 86.66300201 W
P16 1000.00 usec
CNST30 0.598115

F1 - Acquisition parameters
TD 256
SFO1 150.9179 MHz
FIDRES 278.272491 Hz
SW 236.015 ppm
FnMODE Echo-Antiecho

F2 - Processing parameters
SI 2048
SF 600.1300127 MHz
WDW SINE
SSB 4
LB 0 Hz
GB 0
PC 1.40

F1 - Processing parameters
SI 1024
MC2 echo-antiecho
SF 150.9025931 MHz
WDW QSINE
SSB 2
LB 0 Hz
GB 0

HP001

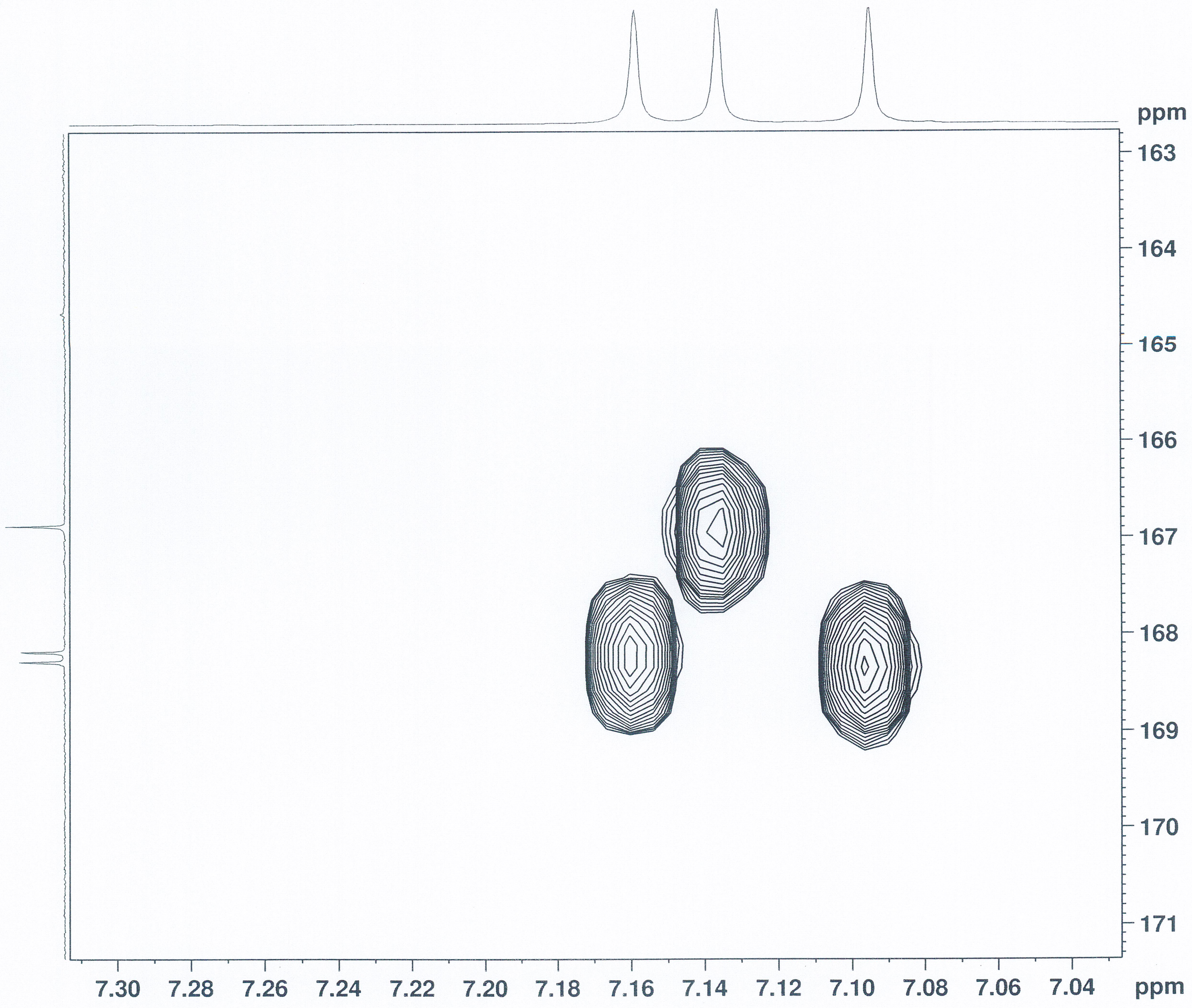

Current Data Parameters		
NAME	Component 3 of Chebulae Fructus_230401	
EXPNO	6	
PROCNO	1	
F2 - Acquisition Parameters		
Date_	20231013	
Time	20.35	h
INSTRUM	Avance	
PROBHD	Z172446_0005 (	
PULPROG	hmbcetgpl3nd	
TD	4096	
SOLVENT	MeOD	
NS	54	
DS	16	
SWH	7812.500	Hz
FIDRES	3.814697	Hz
AQ	0.2621440	sec
RG	101	
DW	64.000	usec
DE	6.50	usec
TE	298.2	K
CNST6	120.0000000	
CNST7	170.0000000	
CNST13	8.0000000	
D0	0.00000300	sec
D1	2.00000000	sec
D6	0.06250000	sec
D16	0.00020000	sec
IN0	0.00001404	sec
TDav	1	
SFO1	600.1330006	MHz
NUC1	1H	
P1	11.92	usec
P2	23.84	usec
PLW1	17.17900085	W
SFO2	150.9178988	MHz
NUC2	13C	
P3	11.80	usec
P24	2000.00	usec
PLW2	86.66300201	W
P16	1000.00	usec
CNST30	0.598115	
F1 - Acquisition parameters		
TD	256	
SFO1	150.9179	MHz
FIDRES	278.272491	Hz
SW	236.015	ppm
FnMODE	Echo-Antiecho	
F2 - Processing parameters		
SI	2048	
SF	600.1300127	MHz
WDW	SINE	
SSB	4	
LB	0	Hz
GB	0	
PC	1.40	
F1 - Processing parameters		
SI	1024	
MC2	echo-antiecho	
SF	150.9025931	MHz
WDW	QSINE	
SSB	2	
LB	0	Hz
GB	0	

HP001

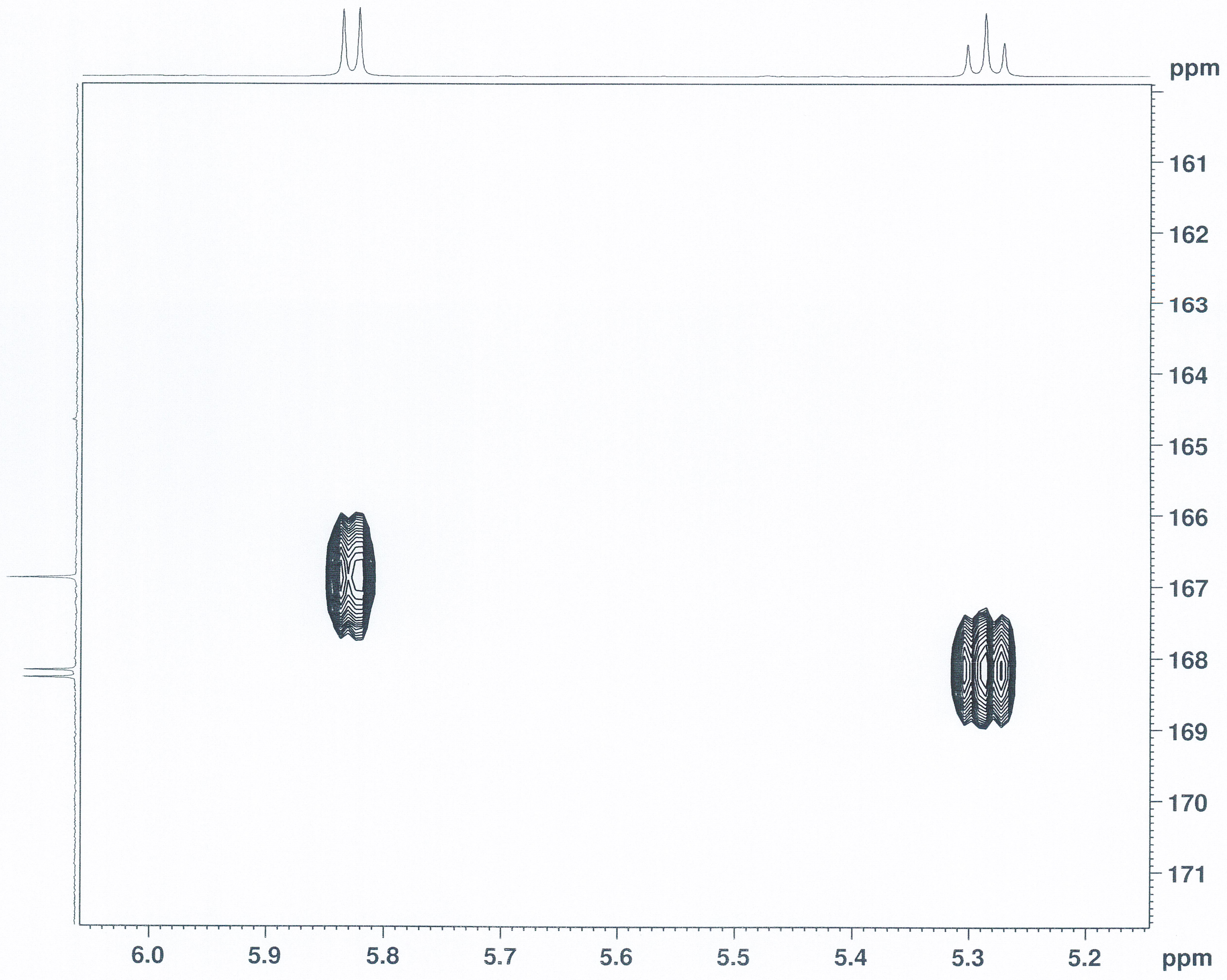

BRUKER

```
Current Data Parameters
NAME      Component 3 of
          Chebulae Fructus_230401
EXPNO                 6
PROCNO                1

F2 - Acquisition Parameters
Date_          20231013
Time              20.35 h
INSTRUM          Avance
PROBHD   Z172446_0005 (
PULPROG    hmbcetgpl3nd
TD                 4096
SOLVENT            MeOD
NS                   54
DS                   16
SWH            7812.500 Hz
FIDRES         3.814697 Hz
AQ            0.2621440 sec
RG                  101
DW               64.000 usec
DE                 6.50 usec
TE                298.2 K
CNST6       120.0000000
CNST7       170.0000000
CNST13        8.0000000
D0           0.00000300 sec
D1           2.00000000 sec
D6           0.06250000 sec
D16          0.00020000 sec
IN0          0.00001404 sec
TDav                  1
SFO1        600.1330006 MHz
NUC1                 1H
P1                11.92 usec
P2                23.84 usec
PLW1        17.17900085 W
SFO2        150.9178988 MHz
NUC2                13C
P3                11.80 usec
P24             2000.00 usec
PLW2        86.66300201 W
P16             1000.00 usec
CNST30         0.598115

F1 - Acquisition parameters
TD                  256
SFO1           150.9179 MHz
FIDRES       278.272491 Hz
SW              236.015 ppm
FnMODE     Echo-Antiecho

F2 - Processing parameters
SI                 2048
SF          600.1300127 MHz
WDW                SINE
SSB                   4
LB         0 Hz
GB         0
PC                 1.40

F1 - Processing parameters
SI                 1024
MC2        echo-antiecho
SF          150.9025931 MHz
WDW               QSINE
SSB                   2
LB         0 Hz
GB         0
```

HP001

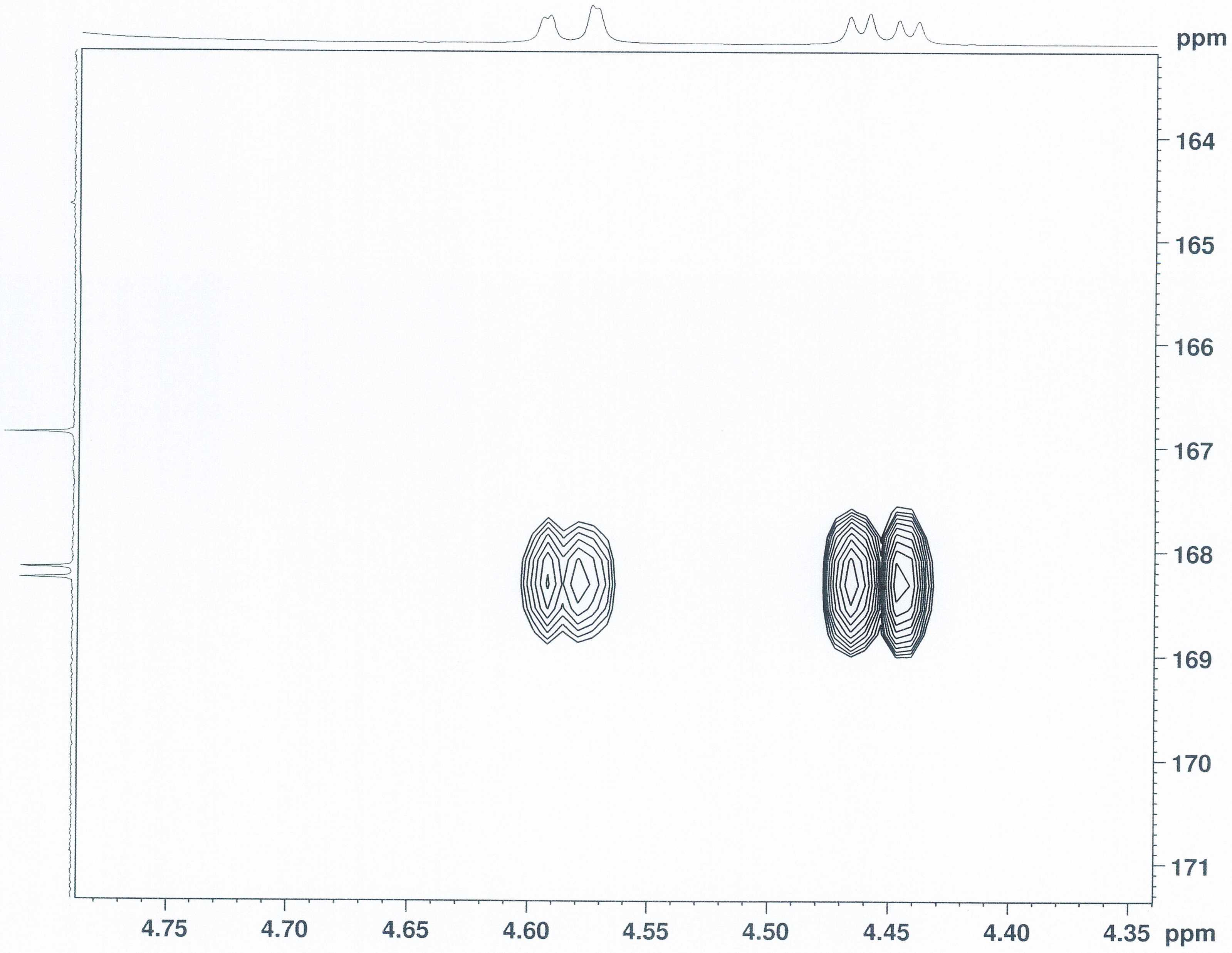

Current Data Parameters		
NAME	Component 3 of Chebulae Fructus_230401	
EXPNO	6	
PROCNO	1	
F2 - Acquisition Parameters		
Date_	20231013	
Time	20.35	h
INSTRUM	Avance	
PROBHD	Z172446_0005 (	
PULPROG	hmbcetgpl3nd	
TD	4096	
SOLVENT	MeOD	
NS	54	
DS	16	
SWH	7812.500	Hz
FIDRES	3.814697	Hz
AQ	0.2621440	sec
RG	101	
DW	64.000	usec
DE	6.50	usec
TE	298.2	K
CNST6	120.0000000	
CNST7	170.0000000	
CNST13	8.0000000	
D0	0.00000300	sec
D1	2.00000000	sec
D6	0.06250000	sec
D16	0.00020000	sec
IN0	0.00001404	sec
TDav	1	
SFO1	600.1330006	MHz
NUC1	1H	
P1	11.92	usec
P2	23.84	usec
PLW1	17.17900085	W
SFO2	150.9178988	MHz
NUC2	13C	
P3	11.80	usec
P24	2000.00	usec
PLW2	86.66300201	W
P16	1000.00	usec
CNST30	0.598115	
F1 - Acquisition parameters		
TD	256	
SFO1	150.9179	MHz
FIDRES	278.272491	Hz
SW	236.015	ppm
FnMODE	Echo-Antiecho	
F2 - Processing parameters		
SI	2048	
SF	600.1300127	MHz
WDW	SINE	
SSB	4	
LB	0	Hz
GB	0	
PC	1.40	
F1 - Processing parameters		
SI	1024	
MC2	echo-antiecho	
SF	150.9025931	MHz
WDW	QSINE	
SSB	2	
LB	0	Hz
GB	0	

HP001

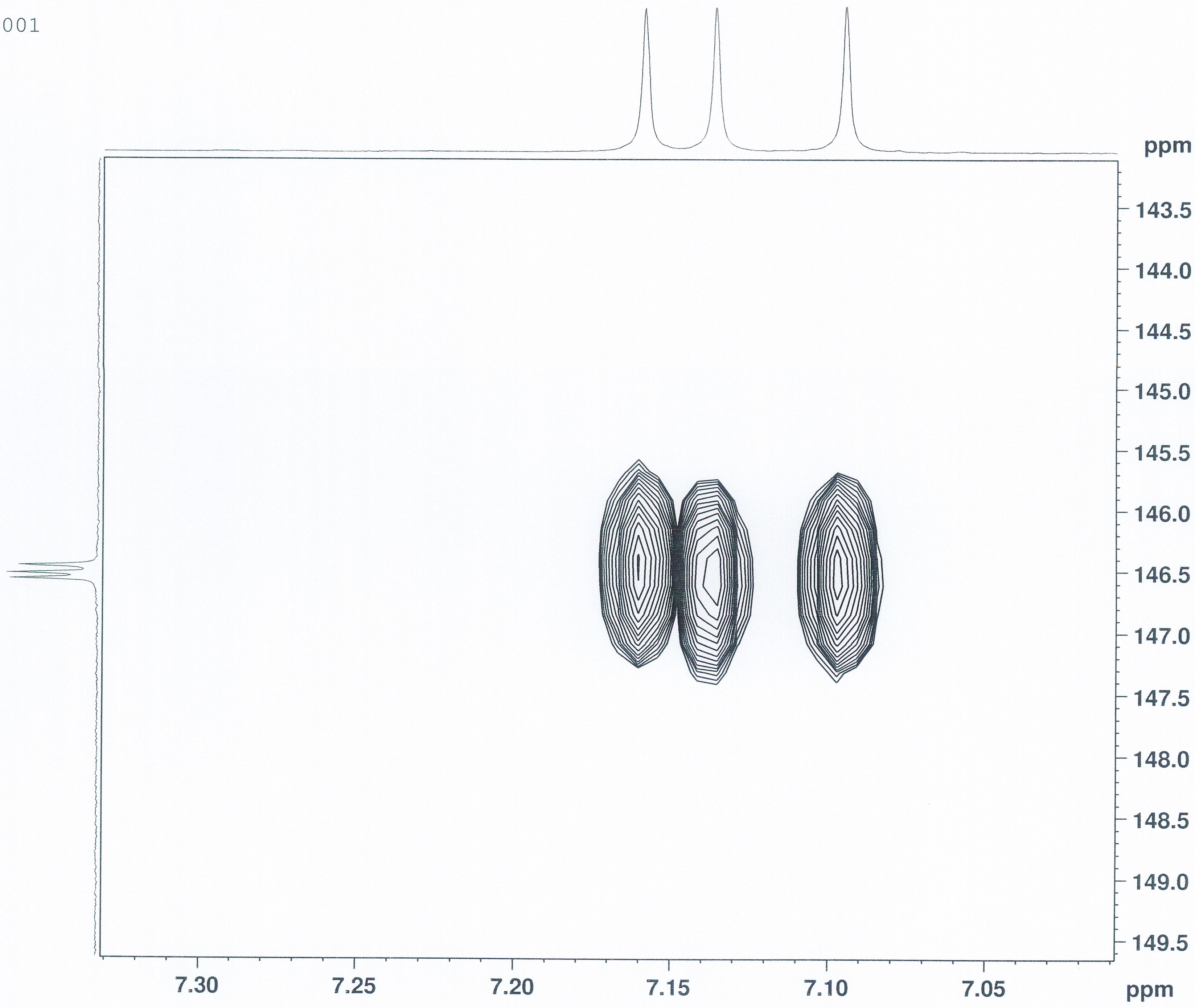


```
Current Data Parameters
NAME       Component 3 of
            Chebulae Fructus_230401
EXPNO                 6
PROCNO                1

F2 - Acquisition Parameters
Date_          20231013
Time              20.35 h
INSTRUM          Avance
PROBHD   Z172446_0005 (
PULPROG    hmbcetgpl3nd
TD                 4096
SOLVENT            MeOD
NS                   54
DS                   16
SWH            7812.500 Hz
FIDRES         3.814697 Hz
AQ            0.2621440 sec
RG                  101
DW               64.000 usec
DE                 6.50 usec
TE                298.2 K
CNST6       120.0000000
CNST7       170.0000000
CNST13        8.0000000
D0           0.00000300 sec
D1           2.00000000 sec
D6           0.06250000 sec
D16          0.00020000 sec
IN0          0.00001404 sec
TDav                  1
SFO1        600.1330006 MHz
NUC1                 1H
P1                11.92 usec
P2                23.84 usec
PLW1        17.17900085 W
SFO2        150.9178988 MHz
NUC2                13C
P3                11.80 usec
P24             2000.00 usec
PLW2        86.66300201 W
P16             1000.00 usec
CNST30         0.598115

F1 - Acquisition parameters
TD                  256
SFO1           150.9179 MHz
FIDRES       278.272491 Hz
SW              236.015 ppm
FnMODE     Echo-Antiecho

F2 - Processing parameters
SI                 2048
SF          600.1300127 MHz
WDW                SINE
SSB                   4
LB         0 Hz
GB         0
PC                 1.40

F1 - Processing parameters
SI                 1024
MC2        echo-antiecho
SF          150.9025931 MHz
WDW               QSINE
SSB                   2
LB         0 Hz
GB         0
```

HP001

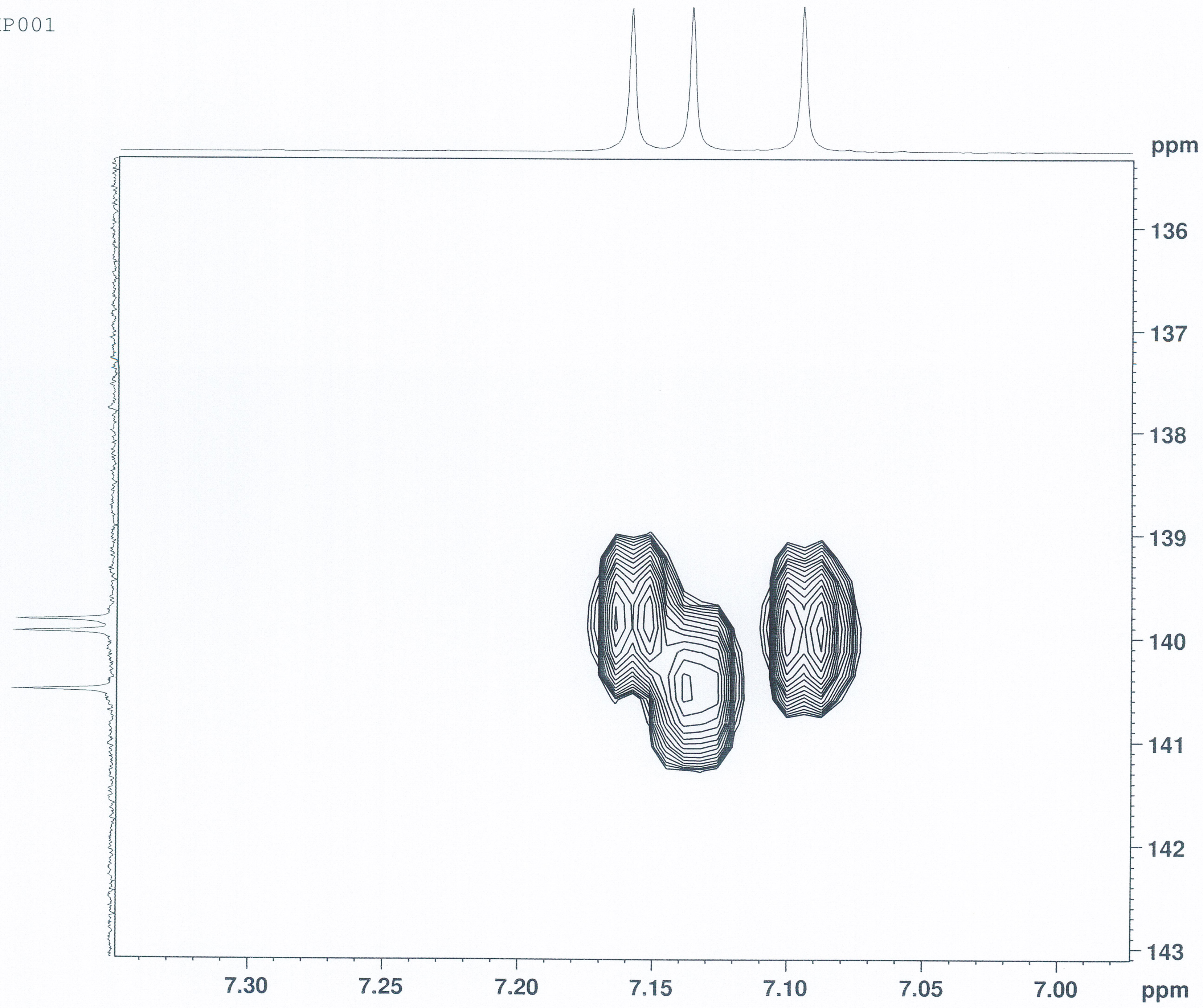

Current Data Parameters
NAME Component 3 of Chebulae Fructus_230401
EXPNO 6
PROCNO 1

F2 - Acquisition Parameters
Date_ 20231013
Time 20.35 h
INSTRUM Avance
PROBHD Z172446_0005 (
PULPROG hmbcetgpl3nd
TD 4096
SOLVENT MeOD
NS 54
DS 16
SWH 7812.500 Hz
FIDRES 3.814697 Hz
AQ 0.2621440 sec
RG 101
DW 64.000 usec
DE 6.50 usec
TE 298.2 K
CNST6 120.0000000
CNST7 170.0000000
CNST13 8.0000000
D0 0.00000300 sec
D1 2.00000000 sec
D6 0.06250000 sec
D16 0.00020000 sec
IN0 0.00001404 sec
TDav 1
SFO1 600.1330006 MHz
NUC1 1H
P1 11.92 usec
P2 23.84 usec
PLW1 17.17900085 W
SFO2 150.9178988 MHz
NUC2 13C
P3 11.80 usec
P24 2000.00 usec
PLW2 86.66300201 W
P16 1000.00 usec
CNST30 0.598115

F1 - Acquisition parameters
TD 256
SFO1 150.9179 MHz
FIDRES 278.272491 Hz
SW 236.015 ppm
FnMODE Echo-Antiecho

F2 - Processing parameters
SI 2048
SF 600.1300127 MHz
WDW SINE
SSB 4
LB 0 Hz
GB 0
PC 1.40

F1 - Processing parameters
SI 1024
MC2 echo-antiecho
SF 150.9025931 MHz
WDW QSINE
SSB 2
LB 0 Hz
GB 0

HP001

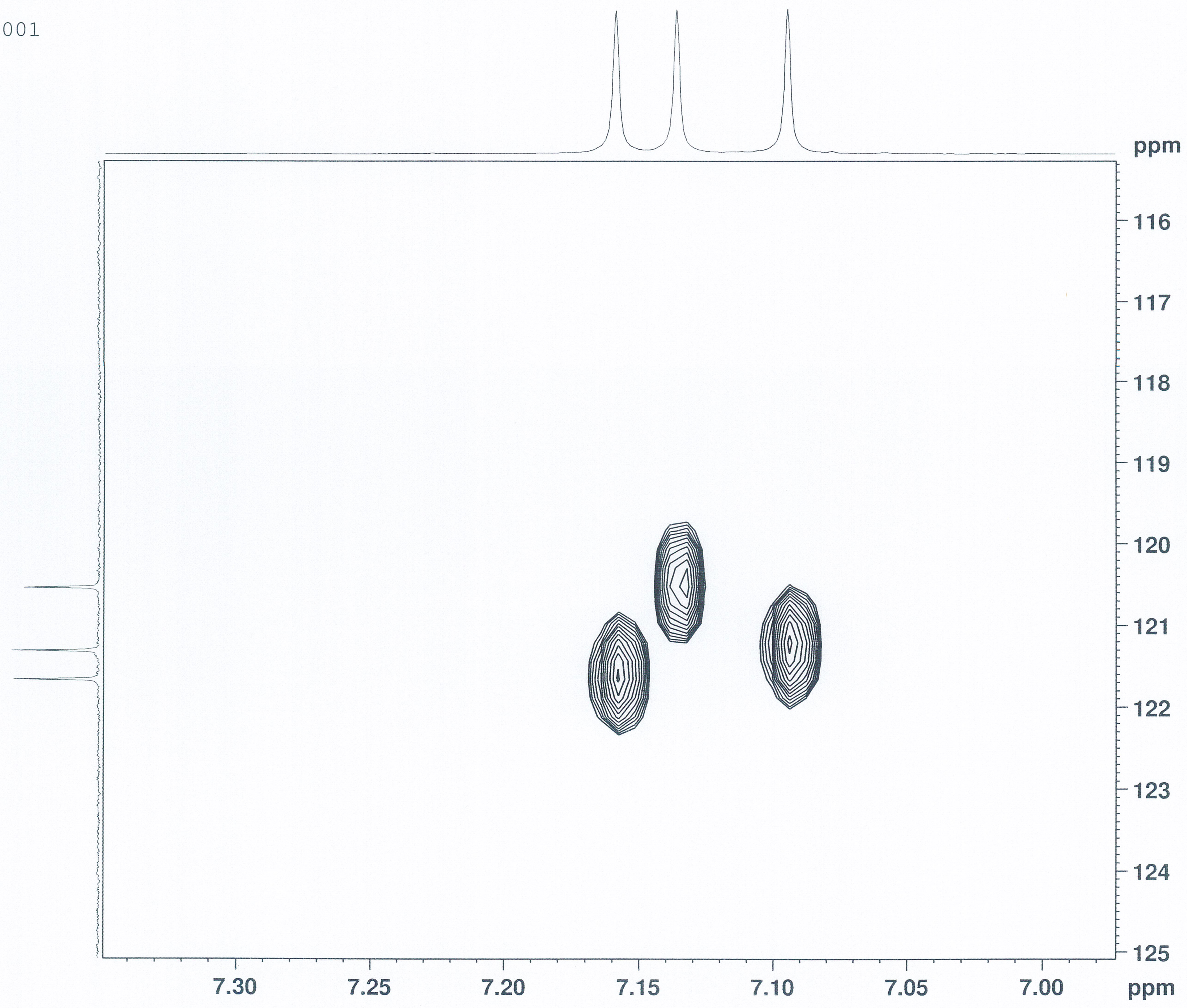


```
Current Data Parameters
NAME      Component 3 of
            Chebulae Fructus_230401
EXPNO                    6
PROCNO                   1

F2 - Acquisition Parameters
Date_             20231013
Time                 20.35 h
INSTRUM             Avance
PROBHD    Z172446_0005 (
PULPROG       hmbcetgpl3nd
TD                    4096
SOLVENT               MeOD
NS                      54
DS                      16
SWH               7812.500 Hz
FIDRES            3.814697 Hz
AQ               0.2621440 sec
RG                     101
DW                  64.000 usec
DE                    6.50 usec
TE                   298.2 K
CNST6          120.0000000
CNST7          170.0000000
CNST13           8.0000000
D0              0.00000300 sec
D1              2.00000000 sec
D6              0.06250000 sec
D16             0.00020000 sec
IN0             0.00001404 sec
TDav                     1
SFO1           600.1330006 MHz
NUC1                    1H
P1                   11.92 usec
P2                   23.84 usec
PLW1           17.17900085 W
SFO2           150.9178988 MHz
NUC2                   13C
P3                   11.80 usec
P24                2000.00 usec
PLW2           86.66300201 W
P16                1000.00 usec
CNST30            0.598115

F1 - Acquisition parameters
TD                     256
SFO1              150.9179 MHz
FIDRES          278.272491 Hz
SW                 236.015 ppm
FnMODE        Echo-Antiecho

F2 - Processing parameters
SI                    2048
SF             600.1300127 MHz
WDW                   SINE
SSB                      4
LB          0 Hz
GB          0
PC                    1.40

F1 - Processing parameters
SI                    1024
MC2          echo-antiecho
SF             150.9025931 MHz
WDW                  QSINE
SSB                      2
LB          0 Hz
GB          0
```

HP001

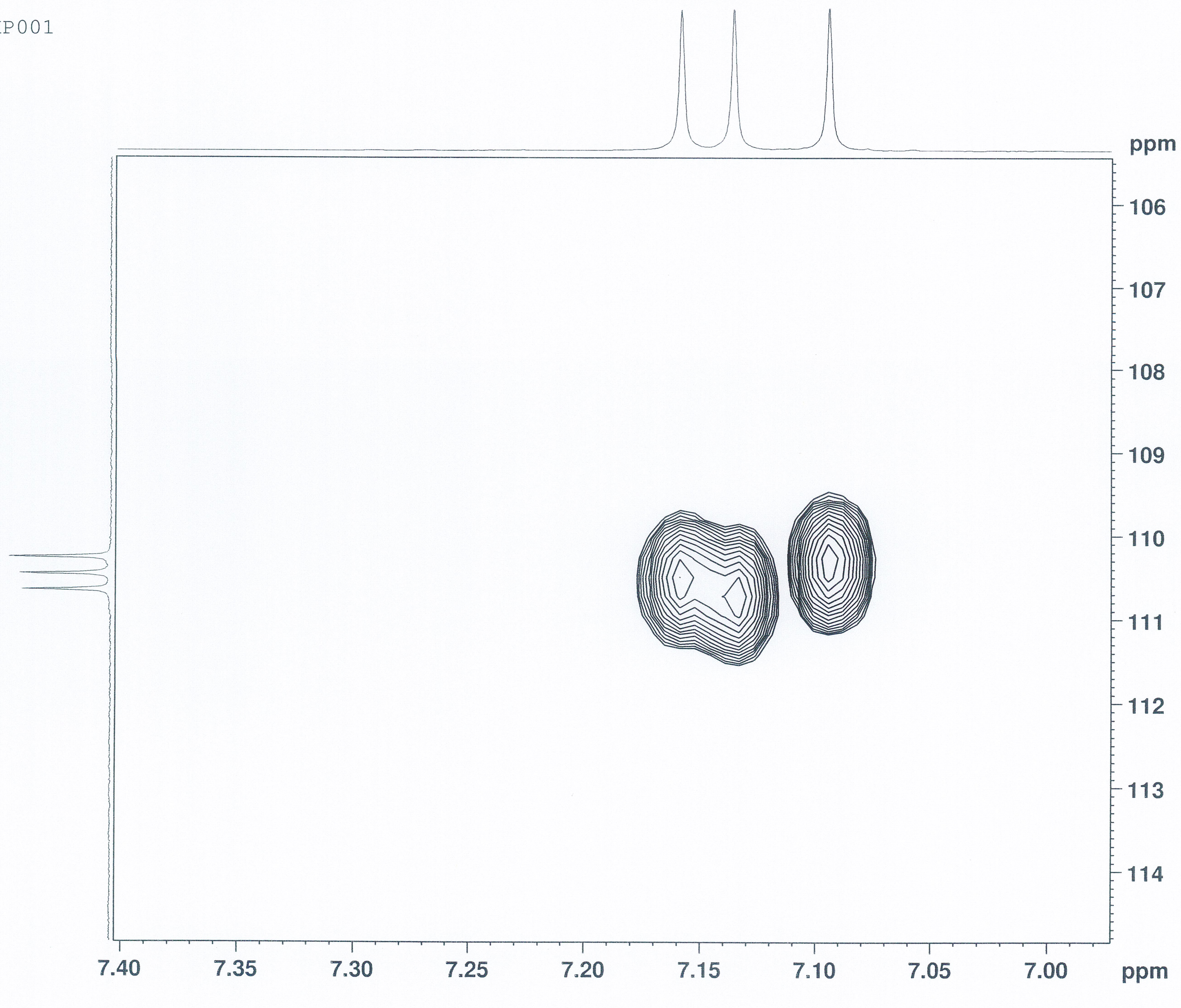


```
Current Data Parameters
NAME      Component 3 of
          Chebulae Fructus_230401
EXPNO                  6
PROCNO                 1

F2 - Acquisition Parameters
Date_           20231013
Time               20.35 h
INSTRUM           Avance
PROBHD    Z172446_0005 (
PULPROG     hmbcetgpl3nd
TD                  4096
SOLVENT             MeOD
NS                    54
DS                    16
SWH             7812.500 Hz
FIDRES          3.814697 Hz
AQ             0.2621440 sec
RG                   101
DW                64.000 usec
DE                  6.50 usec
TE                 298.2 K
CNST6        120.0000000
CNST7        170.0000000
CNST13         8.0000000
D0            0.00000300 sec
D1            2.00000000 sec
D6            0.06250000 sec
D16           0.00020000 sec
IN0           0.00001404 sec
TDav                   1
SFO1        600.1330006 MHz
NUC1                  1H
P1                 11.92 usec
P2                 23.84 usec
PLW1         17.17900085 W
SFO2        150.9178988 MHz
NUC2                 13C
P3                 11.80 usec
P24              2000.00 usec
PLW2         86.66300201 W
P16              1000.00 usec
CNST30          0.598115

F1 - Acquisition parameters
TD                   256
SFO1            150.9179 MHz
FIDRES        278.272491 Hz
SW               236.015 ppm
FnMODE       Echo-Antiecho

F2 - Processing parameters
SI                  2048
SF           600.1300127 MHz
WDW                 SINE
SSB                    4
LB          0 Hz
GB          0
PC                  1.40

F1 - Processing parameters
SI                  1024
MC2         echo-antiecho
SF           150.9025931 MHz
WDW                QSINE
SSB                    2
LB          0 Hz
GB          0
```

HP001

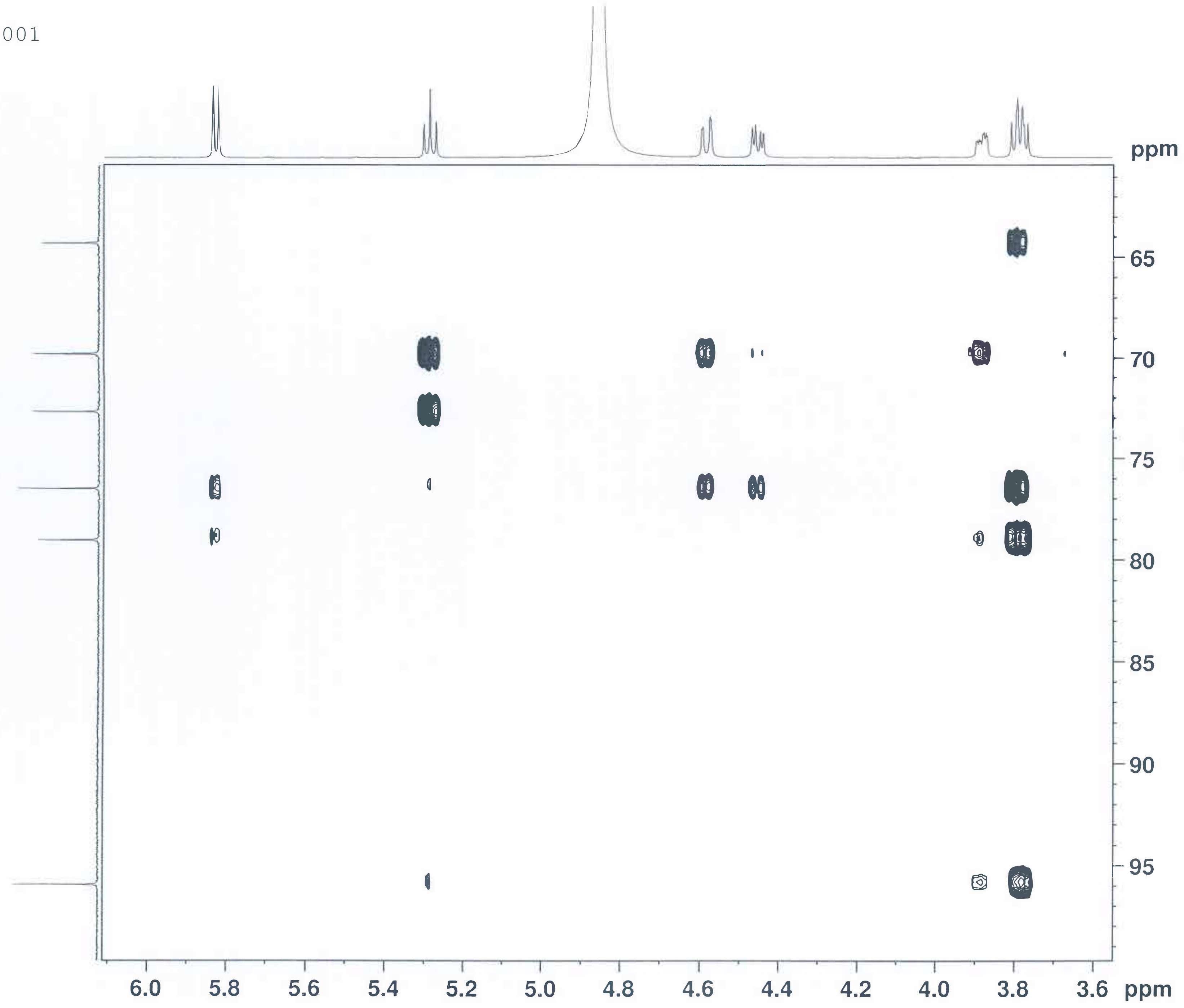

BRUKER

```
Current Data Parameters
NAME        Component 3 of
            Chebulae Fructus_23040[illegible]
EXPNO                     6
PROCNO                    1

F2 - Acquisition Parameters
Date_              20231013
Time                  20.35 h
INSTRUM              Avance
PROBHD       Z172446_0005 (
PULPROG        hmbcetgpl3nd
TD                     4096
SOLVENT                MeOD
NS                       54
DS                       16
SWH                7812.500 Hz
FIDRES             3.814697 Hz
AQ                0.2621440 sec
RG                      101
DW                   64.000 usec
DE                     6.50 usec
TE                    298.2 K
CNST6           120.0000000
CNST7           170.0000000
CNST13            8.0000000
D0               0.00000300 sec
D1               2.00000000 sec
D6               0.06250000 sec
D16              0.00020000 sec
IN0              0.00001404 sec
TDav                      1
SFO1            600.1330006 MHz
NUC1                     1H
P1                    11.92 usec
P2                    23.84 usec
PLW1            17.17900085 W
SFO2            150.9178988 MHz
NUC2                    13C
P3                    11.80 usec
P24                 2000.00 usec
PLW2            86.66300201 W
P16                 1000.00 usec
CNST30             0.598115

F1 - Acquisition parameters
TD                      256
SFO1               150.9179 MHz
FIDRES           278.272491 Hz
SW                  236.015 ppm
FnMODE        Echo-Antiecho

F2 - Processing parameters
SI                     2048
SF              600.1300127 MHz
WDW                    SINE
SSB                       4
LB          0 Hz
GB          0
PC                     1.40

F1 - Processing parameters
SI                     1024
MC2          echo-antiecho
SF              150.9025931 MHz
WDW                   QSINE
SSB                       2
LB          0 Hz
GB          0
```
